# Light generation of intracellular Ca^2+^ signals by a genetically encoded protein BACCS

**DOI:** 10.1038/ncomms9021

**Published:** 2015-08-18

**Authors:** Tomohiro Ishii, Koji Sato, Toshiyuki Kakumoto, Shigenori Miura, Kazushige Touhara, Shoji Takeuchi, Takao Nakata

**Affiliations:** 1 Department of Cell Biology, Graduate School of Medical and Dental Science, Tokyo Medical and Dental University, 1-5-45 Yushima, Bunkyo-ku, Tokyo 113-8510, Japan; 2 The Center for Brain Integration Research, Tokyo Medical and Dental University, 1-5-45 Yushima, Bunkyo-ku, Tokyo 113-8510, Japan; 3 Division of Bioregulatory Signaling, Okazaki Institute for Integrative Bioscience, 5-1 Higashiyama, Myodaijichou, Okazaki, Aichi 444-8585, Japan; 4 Institute of Industrial Science, The University of Tokyo, 4-6-1 Komaba, Meguro-ku, Tokyo 153-8505, Japan; 5 ERATO Takeuchi Biohybrid Innovation Project, Japan Science and Technology Agency, Tokyo 153-8904, Japan; 6 Department of Applied Biological Chemistry, Graduate School of Agricultural and Life Sciences, The University of Tokyo, 1-1-1 Yayoi, Bunkyo-ku, Tokyo 113-8657, Japan; 7 ERATO Touhara Chemosensory Signal Project, JST, The University of Tokyo, 1-1-1 Yayoi, Bunkyo-ku, Tokyo 113-8657, Japan

## Abstract

Ca^2+^ signals are highly regulated in a spatiotemporal manner in numerous cellular physiological events. Here we report a genetically engineered blue light-activated Ca^2+^ channel switch (BACCS), as an optogenetic tool for generating Ca^2+^ signals. BACCS opens Ca^2+^-selective ORAI ion channels in response to light. A BACCS variant, dmBACCS2, combined with *Drosophila* Orai, elevates the Ca^2+^ concentration more rapidly, such that Ca^2+^ elevation in mammalian cells is observed within 1 s on light exposure. Using BACCSs, we successfully control cellular events including NFAT-mediated gene expression. In the mouse olfactory system, BACCS mediates light-dependent electrophysiological responses. Furthermore, we generate BACCS mutants, which exhibit fast and slow recovery of intracellular Ca^2+^. Thus, BACCSs are a useful optogenetic tool for generating temporally various intracellular Ca^2+^ signals with a large dynamic range, and will be applicable to both *in vitro* and *in vivo* studies.

## Abstract (web summary)

Current tools for optogenetic control of intracellular calcium signals currently suffer from slow response time or low dynamic range. Here the authors develop blue light-activated Ca2^+^ channel switch (BACCS) that modulates the activity of Ca2^+^-sensitive Orai channels with high temporal resolution and large dynamic range.

## Graphical abstract

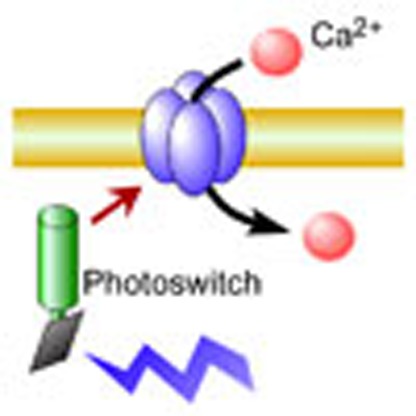

Precise spatial and temporal coordination of molecular events are the basis of many cellular functions. Recently, there has been growing interest in using optogenetic tools to investigate cellular functions, because light is non-invasive and can be easily controlled spatiotemporally. Optical control enables precise regulation of intracellular signals in the target cells or even in the local area of single cells^1^,^2^. The most successful optogenetic tool, channelrhodopsin (ChR) from the green alga *Chlamydomonas reinhardtii*, modulates the membrane voltage in response to light and has been widely applied in the field of neurosciences^3^. More recently, in addition to natural photosensory proteins, there have been advances in the development of genetically encoded photosensory modules for investigating cellular functions. One such photosensory module is the *Avena sativa* (oat) LOV2-Jα domain^4^,^5^, which has been fused with various effector domains of proteins to generate novel engineered light-controlled molecules^2^,^6^,^7^,^8^.

Ca^2+^ is a ubiquitous second messenger in nearly all cells and regulates a wide variety of cell functions from cell division to cell death, including gene expression, cell migration, secretion, neural activities and muscle contraction. Ca^2+^ signals function over a wide timescale, from milliseconds for synaptic vesicle release to hours for gene expression leading to cell development and differentiation^9^,^10^. Furthermore, Ca^2+^ signals play important roles at the subcellular level, such as in learning and memory at spiny dendrites and in neurotransmitter release at synaptic endings in a single neuron^11^. A genetically encoded light-activated protein that regulates intracellular Ca^2+^ signals could be useful and has been eagerly awaited^12^,^13^.

Compared with caged compounds^14^, genetically encoded light-sensitive proteins are more convenient to apply *in vivo*, because they usually do not require an external supply of chromophores to absorb photons^15^. ChR2 (refs 16, 17), opto-α1AR^18^, melanopsin^19^, LOVS1K^20^ and PACR^21^ are currently available optogenetic tools to generate intracellular Ca^2+^ signals. ChR2 is a light-activated cation channel^16^, while opto-α1AR and melanopsin stimulate the phospholipase C cascade, thereby indirectly regulating Ca^2+^ signals. Therefore, these optogenetic tools are not specific for Ca^2+^ signals. LOVS1K is a fusion protein of a plant LOV2-Jα domain and a human stromal interaction molecule-1 (STIM1) fragment, and is a light-dependent regulator of Ca^2+^-selective ORAI1 channels^22^. Repeated photoactivation of LOVS1K co-expressed with ORAI1 was reported to induce a slow increase in global Ca^2+^, reaching an intracellular Ca^2+^ concentration of ∼500 nM after 40 min^20^. PACR is a Ca^2+^-binding protein with an inserted LOV2-Jα domain, and releases Ca^2+^ in response to light. The amplitude of Ca^2+^ elevation by PACR is relatively small, and requires improvement to control many cellular events^21^. Engineering of an optogenetic tool that can control intracellular Ca^2+^ signals with high selectivity, good time resolution and large dynamic range is required for practical use.

Here we develop an optogenetic tool, BACCS, to generate intracellular Ca^2+^ signals. One of the BACCS variants induces an increase in intracellular global Ca^2+^ signals within 1 s. BACCS is tuneable for its recovery kinetics, enabling fast and slow recovery of intracellular Ca^2+^. We further demonstrate the application of BACCS *in vitro* and in *ex vivo* whole-mount preparations. Thus, BACCS is a useful optogenetic tool for regulating a wide variety of cellular events via intracellular Ca^2+^ signals in various cell types both *in vitro* and *in vivo*.

## Results

### Engineering of a photoswitch for a Ca^2+^-selective channel

**Figure 1:**
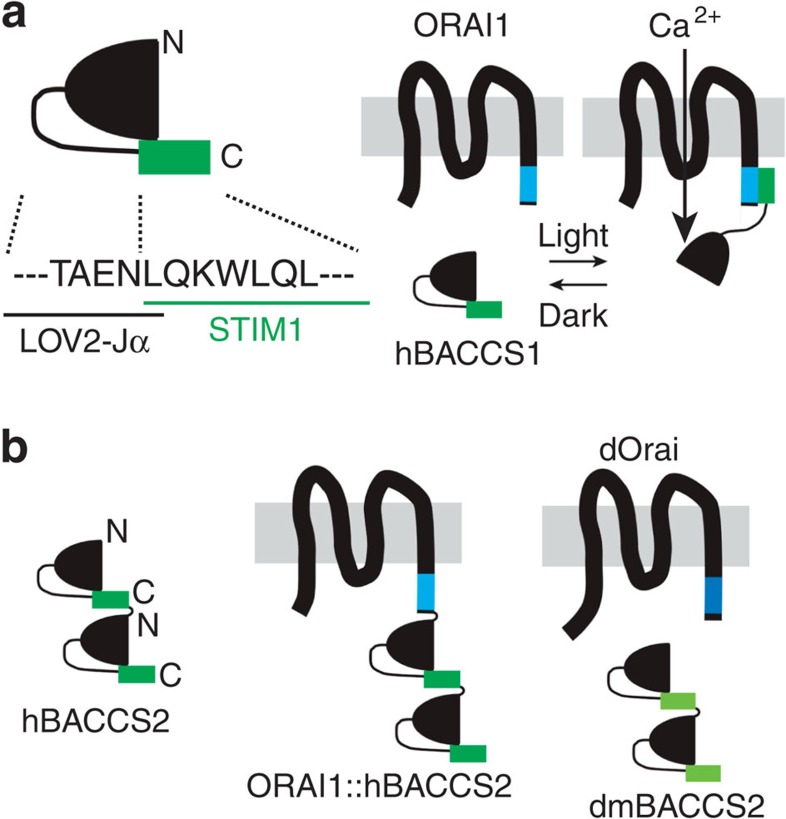
Schematic representation of BACCSs. (**a**) Schematic design of hBACCS1. In the dark, the interaction of hBACCS1 and ORAI1 is inhibited by steric hindrance of the STIM1 effector domain. On blue light exposure, a conformational change of LOV2-Jα exposes the STIM1 effector domain to ORAI1 channels, resulting in an influx of extracellular Ca^2+^. N, N terminus; C, C terminus. (**b**) Schematic design of BACCS variants: hBACCS2, a tandem dimer of hBACCS1; ORAI1::hBACCS2, a fusion protein of ORAI1 and hBACCS2; dmBACCS2, a BACCS2 protein constructed using the corresponding signalling domain of *Drosophila* Stim; dOrai, *Drosophila* Orai.

We generated a series of fusion genes of the LOV2-Jα sequence from *A. sativa* phototropin 1 (refs 4, 5) as a photosensory module and the regulatory sequence for ORAI1 from human STIM1 (refs 23, 24, 25, 26) as a signal effector (Fig. 1a; Supplementary Fig. 1). We assumed that the closer the photosensor and the signal effector were, the more efficient steric hindrance of the STIM1 interaction would be, thereby enabling inhibition of the signalling function in the dark. The basic core unit of BACCS was selected by combining the following three screening steps (see Supplementary Fig. 1 for details). First, the minimal signal effector domain of the STIM1 fragment for ORAI1 activation was defined. Second, the minimal STIM1 fragment was fused to the C terminus of a deletion series of LOV2-Jα to find the structure causing steric hindrance of the STIM1 interaction by LOV2-Jα. Third, the candidate fusion proteins were expressed with nuclear factor of activated T cells (NFAT)::CFP (‘::' represents a fusion) to examine light-induced NFAT translocation from the cytoplasm to the nucleus. It has been reported that elevation of intracellular Ca^2+^ (>200–300 nM) induces dephosphorylation of NFAT, followed by its nuclear translocation^27^. LOV2-Jα (amino acids 404–538)::STIM1 (amino acids 347–448) was the most efficient photoswitch, displaying high sensitivity and low basal activity, and is designated human blue light-activated Ca^2+^ channel switch 1 (hBACCS1). hBACCS1 has the structural feature that a leucine residue (originally an isoleucine in LOV2-Jα) at the junction of the fusion protein, which is important for the function of both LOV2-Jα and STIM1, is shared between them (Fig. 1a). Therefore, the dark-state form of LOV2-Jα will block the function of STIM1 through steric hindrance over the leucine residue. Three variants of BACCS were designed (Fig. 1b): (1) hBACCS2, a dimer of hBACCS1; (2) ORAI1::hBACCS2, a fusion protein of human ORAI1 and hBACCS2; and (3) dmBACCS2, protein with the same structure as hBACCS2 except that *Drosophila* Stim was used instead of human STIM1. The designs of hBACCS2 and ORAI1::hBACCS2 were based on the observation that a tandem dimer of STIM1 (336–485) and its ORAI1 fusion protein can efficiently open ORAI1 channels^28^.

### Calcium response of ORAI1 activated by hBACCS

**Figure 2:**
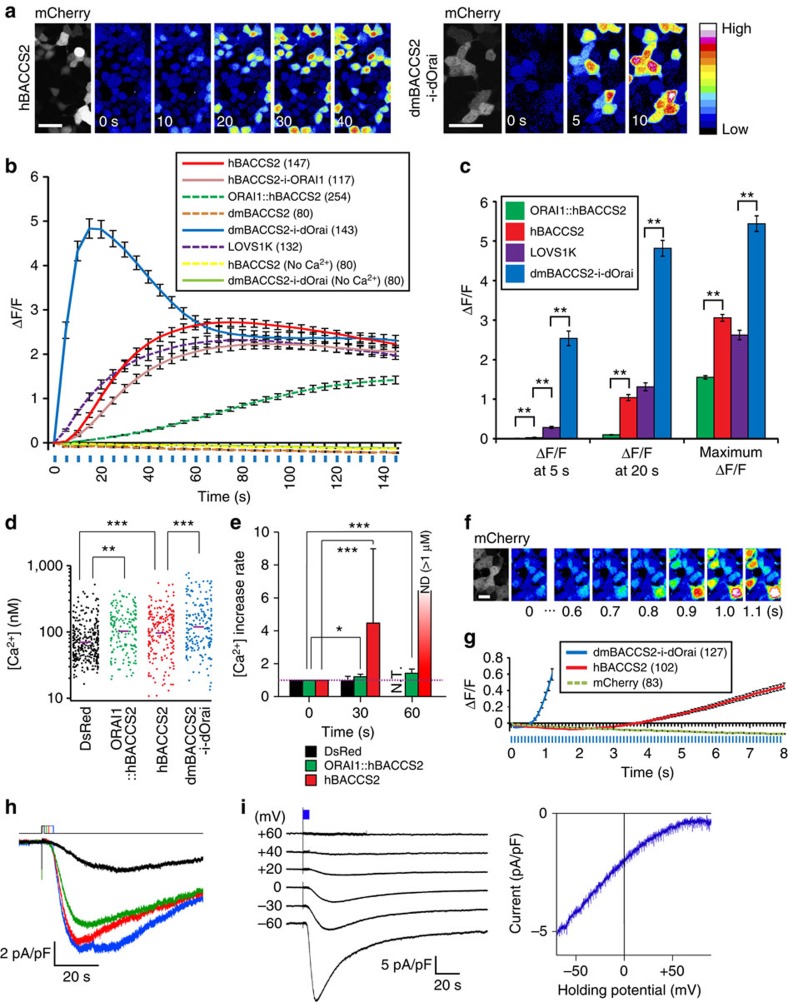
Characterization of BACCSs expressed in HEK293T cells. (**a**) Representative Ca^2+^ responses in cells transiently expressing *hBACCS2-IRES-mCherry* or *dmBACCS2-IRES-dOrai-IRES-mCherry* with Fluo-8/AM during the course of photoactivation (488-nm laser; 0.2 Hz). Pseudocoloured images are shown. A key for the heatmap is on the right. Scale bar, 40 μm. (**b**) Time course of Fluo-8/AM fluorescence changes in cells transiently expressing various constructs in the presence or absence of extracellular Ca^2+^ during the course of photoactivation (488-nm laser; 0.2 Hz; illumination indicated by blue vertical lines). i, *IRES*. (**c**) Mean Fluo-8/AM fluorescence changes at 5 and 20 s, and mean maximum fluorescence changes in each cell during the course of photoactivation in **b**. Significance was assessed by *t*-test. (**d**) Basal intracellular Ca^2+^ concentrations in cells expressing *DsRed* (*n*=313), *ORAI1::hBACCS2-IRES-mem::tdTomato* (*n*=163), *hBACCS2-IRES-mem::tdTomato* (*n*=201) or *dmBACCS2-IRES-dOrai-IRES-mem::tdTomato* (*n*=181). Purple bars indicate geometric means. Significance was assessed by analysis of variance and Fisher's PLSD test. (**e**) Rates of [Ca^2+^] increase. The times after ultraviolet illumination for BACCS activation are indicated. Because of high fluorescence signals beyond the Fura-2/AM detection limit (>1 μM) immediately after illumination, dmBACCS2 is not included. Data represent means±s.d. Significance was assessed by *t*-test. (**f**) Images of Fluo-8/AM responses in HEK293T cells transiently expressing *dmBACCS2-IRES-dOrai-IRES-mCherry* during the course of photoactivation (488 nm; 10 Hz). Pseudocoloured images are shown as in **a**. Scale bar, 20 μm. (**g**) Time course of Fluo-8/AM fluorescence changes in HEK293T cells transiently expressing *dmBACCS2-IRES-dOrai-IRES-mCherry*, *hBACCS2-IRES-mCherry* or *mCherry* during the course of photoactivation (488 nm; 10 Hz; blue vertical lines). (**h**,**i**) Whole-cell inward current responses in HEK293T cells expressing *dmBACCS2-IRES-dOrai*. (**h**) Current responses with LED blue light stimulation of increasing duration as indicated by different colours (1, 2, 3 and 5 s) at a holding potential of −60 mV. (**i**) Current responses of HEK293T cells at various holding potentials (left) and I–V relationships obtained by a ramp protocol. The blue bar indicates the timing of blue light (470 nm) stimulation. Data in **b**,**c** and **g** represent means±s.e.m. The cell numbers analysed in **b** and **g** are indicated in parentheses. All data were obtained from more than three independent experiments. Significance: *P<0.05, **P<0.01, ***P<0.001.

To evaluate the hBACCS function, we performed Ca^2+^ imaging in HEK293T cells expressing *hBACCS2* (Fig. 2a). The widespread expression of ORAI proteins in most tissues^29^,^30^,^31^ will enable the omission of additional introduction of ORAI proteins in most cell types. We compared the following four constructs: *hBACCS1*, *hBACCS2*, *hBACCS2-IRES-ORAI1* and *ORAI1::hBACCS2* (Fig. 2b,c; Supplementary Fig. 2a,b). The *IRES* sequence allows co-translation of hBACCS2 with ORAI1 from a bicistronic messenger RNA. More than 98% of cells expressing *hBACCS2* or *ORAI1::hBACCS2* showed a Ca^2+^ increase on light exposure, while only 46% of cells expressing *hBACCS1* showed an increase (Supplementary Fig. 2a; Supplementary Table 1), suggesting that hBACCS2 and ORAI1::hBACCS2 are more efficient photoswitches than hBACCS1. The time courses of the intracellular Ca^2+^ increase revealed that hBACCS2 induced a faster intracellular Ca^2+^ increase than ORAI1::hBACCS2 (Fig. 2b,c). Exogenous expression of ORAI1 in addition to hBACCS2 (*hBACCS2-IRES-ORAI1*) did not enhance the Ca^2+^ response in HEK293T cells compared with expression of hBACCS2 alone (Fig. 2b). Two ORAI1::hBACCS2 variants with shorter and longer linkers were also examined, resulting in no apparent improvement in Ca^2+^ response (Supplementary Fig. 2c). Removal of Ca^2+^ from the extracellular Ringer's solution prevented the light-induced increase in intracellular Ca^2+^ in cells expressing *hBACCS2* (Fig. 2b), suggesting that the Ca^2+^ increase occurs through influx of extracellular Ca^2+^, rather than release of Ca^2+^ from intracellular organelles such as the endoplasmic reticulum or mitochondria. The Ca^2+^ indicator dye Fura-2/AM was used to measure the intracellular Ca^2+^ concentrations. The geometric means of the basal Ca^2+^ level in cells expressing *ORAI1::hBACCS2* or *hBACCS2* in the dark were both around 100 nM, slightly higher than that in control cells but within the range of physiological basal intracellular Ca^2+^ concentrations (Fig. 2d). In cells expressing *hBACCS2*, the intracellular Ca^2+^ concentration was elevated ∼4.5-fold after 30 s and to beyond 1 μM (>10-fold) after 60 s in response to ultraviolet light (Fig. 2e). The efficiency of photoactivation of hBACCS2 was light intensity dependent. The magnitude of the Ca^2+^ response increased with increasing intensity in the range 0.6–180 μW mm^−2^ of continuous 470-nm light emitting diode (LED) illumination (Supplementary Fig. 2d). Activation of hBACCS2 was reversible (Supplementary Fig. 2e), and repeated photoactivation resulted in similar levels of Ca^2+^ elevation each time (Supplementary Fig. 2f).

Using whole-cell patch-clamp recording, inward currents were recorded at a holding potential of −60 mV in HEK293T cells expressing *ORAI1::hBACCS2* or *hBACCS2* on blue light exposure (Supplementary Fig. 3a,c). Inward currents were also recorded in Jurkat cells (Supplementary Fig. 3e), which are known to have significant endogenous Ca^2+^ release-activated Ca^2+^ currents. The inward rectification of the light-induced current (Supplementary Fig. 3b,d,f) is consistent with the characteristic of ORAI1 channels^32^,^33^,^34^,^35^. Furthermore, photostimulation did not generate outward currents representing efflux of Na^+^ or K^+^ in depolarized HEK293T cells expressing hBACCS2 (Supplementary Fig. 3d), suggesting that hBACCS2 does not activate non-selective cation channels, such as Trpc (transient receptor potential canonical) channels, which give large outward cationic currents at depolarized potential^36^, and that Ca^2+^ is the only charge carrier of photoactivated currents under the whole-cell current recording condition. These results provide further indications that light-activated hBACCS2 can open ORAI channels in the plasma membrane and induce influx of extracellular Ca^2+^.

### Characterization of dmBACCS2

To examine the applicability of hBACCS2 to other species, we performed Ca^2+^ imaging in *Drosophila* S2 cells expressing *hBACCS2*. However, we found no intracellular Ca^2+^ increase on photoactivation (Supplementary Fig. 4a), suggesting that interaction between hBACCS2 and *Drosophila* Orai (dOrai) was absent or insufficient. Since currents through a store-operated Ca^2+^ channel were recorded^37^ and *dOrai* was identified as a gene involved in store-operated Ca^2+^ entry in S2 cells by genome-wide RNA interference screens^33^,^34^,^38^, *dOrai* should be expressed in S2 cells. Therefore, we performed Ca^2+^ imaging in S2 cells expressing *dmBACCS2*, a *Drosophila* version of *BACCS2*, and found that intracellular Ca^2+^ signals were generated in a subset of cells after stimulation with a 488-nm laser (Supplementary Fig. 4b). The time course of the Ca^2+^ changes in S2 cells revealed that the concentration of intracellular Ca^2+^ reached its maximum ∼50 s after illumination (Supplementary Fig. 4c). When Ca^2+^ imaging was performed in HEK293T cells expressing *dmBACCS2* alone, intracellular Ca^2+^ was not elevated by photoactivation (Fig. 2b), suggesting that dmBACCS2 does not activate human ORAI1. As dmBACCS2 appeared to activate dOrai according to the Ca^2+^ imaging experiments in S2 cells, we examined HEK293T cells expressing *dmBACCS2-IRES-dOrai*. Surprisingly, activation of dmBACCS2 in dOrai-expressing HEK293T cells caused fast and robust intracellular Ca^2+^ elevation (Fig. 2a–c). Removal of Ca^2+^ from the extracellular Ringer's solution prevented the light-induced increase in intracellular Ca^2+^ in these cells (Fig. 2b), suggesting that the Ca^2+^ increase occurred through influx of extracellular Ca^2+^. Intracellular Ca^2+^ elevation was observed in 98.0% of cells expressing *dmBACCS2-IRES-dOrai* (Supplementary Table 1). To closely observe the Ca^2+^ changes, the cells were photoactivated with a 488-nm laser and observed every 0.1 s. Intracellular Ca^2+^ elevation was clearly observed within 1 s (Fig. 2f,g). Activation of dmBACCS2 was reversible, and repeated activation resulted in similar levels of intracellular Ca^2+^ elevation each time (Supplementary Fig. 2e,f). We performed whole-cell patch-clamp recording in HEK293T cells expressing *dmBACCS2-IRES-dOrai*, and confirmed the stimulus-dependent response amplitude and the inward rectification (Fig. 2h,i), consistent with the electrical characteristics of store-operated calcium channels in S2 cells^37^. LOVS1K (ref. 20), which is composed of LOV2-Jα (404–546) and STIM1 (233–450) with mRFP, was also analysed for comparison. Photoactivation of LOVS1K in HEK293T cells resulted in slightly faster intracellular Ca^2+^ elevation compared with that due to hBACCS2 in our system (Fig. 2b). However, the speed of the Ca^2+^ elevation and amplitude of the Ca^2+^ increase by dmBACCS2 were significantly greater than those induced by LOVS1K or hBACCS2 (Fig. 2c). The geometric mean of the basal Ca^2+^ level in cells expressing *dmBACCS2-IRES-dOrai* was 119.8 nM (Fig. 2d), higher than that in control cells but almost within the physiological range. It tended to be elevated with increases in the expression level (Supplementary Fig. 5a). We also examined NFAT::GFP subcellular localization in the dark, an alternative physiological assessment of the basal intracellular Ca^2+^ level (Supplementary Fig. 5b,c). We observed that NFAT::GFP was distributed in the cytoplasm, but not in the nucleus, in cells expressing *hBACCS2* or *dmBACCS2-IRES-dOrai* in the dark, indicating a low basal intracellular Ca^2+^ level. In contrast, a large population (64.8%) of cells expressing *LOVS1K* showed nuclear localization of NFAT::GFP without light illumination, suggesting that the basal Ca^2+^ concentration in cells expressing *LOVS1K* was high.

### Generation of Ca^2+^ signals in various cell types

**Figure 3:**
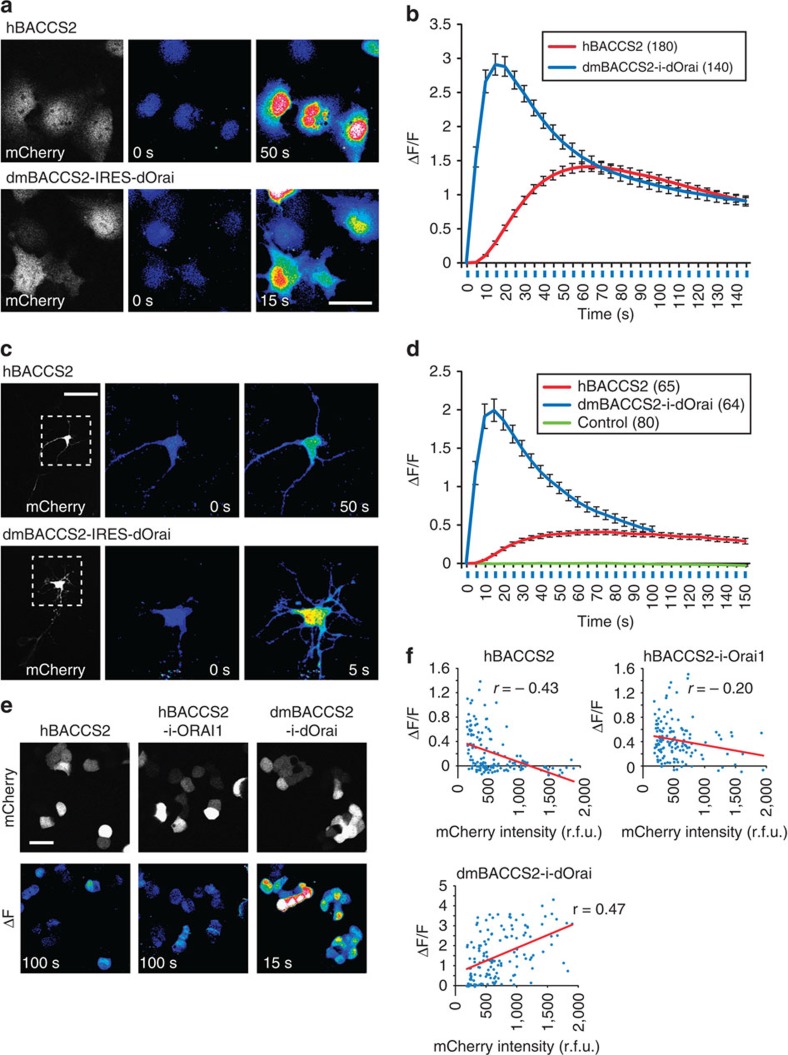
Activation of hBACCS2 and dmBACCS2 in various cell types. Cells were illuminated with a blue laser (488-nm; 0.2 Hz; illumination indicated by blue vertical lines). (**a**) Representative Ca^2+^ responses in Fluo-8/AM-loaded COS-7 cells transiently expressing *hBACCS2-IRES-mCherry* or *dmBACCS2-IRES-dOrai-IRES-mCherry*. Scale bar, 50 μm. (**b**) Time course of Fluo-8/AM fluorescence changes in **a** during the course of photoactivation. Ca^2+^ elevation was observed in 94.7 and 79.5% of cells expressing *hBACCS2* and *dmBACCS2-IRES-dOrai*, respectively. (**c**) Representative Ca^2+^ responses in mouse hippocampal neurons transiently expressing *hBACCS2-IRES-mCherry* or *dmBACCS2-IRES-dOrai-IRES-mCherry*. Fluo-8/AM fluorescence images corresponding to the boxed areas in the left panels are shown. Scale bar, 50 μm. (**d**) Time course of Fluo-8/AM fluorescence changes in the hippocampal neurons in **c** during the course of photoactivation. Cells transiently expressing *mCherry* were used as a negative control. Ca^2+^ elevation was observed in 86.7 and 95.5% of cells expressing *hBACCS2* and *dmBACCS2-IRES-dOrai*, respectively. (**e**) Representative Ca^2+^ responses in Fluo-8/AM-loaded hamster pancreatic β-cell line HIT-T15 cells transiently expressing *hBACCS2-IRES-mCherry*, *hBACCS2-IRES-ORAI1-IRES-mCherry* or *dmBACCS2-IRES-dOrai-IRES-mCherry*. Images of the fluorescence changes (ΔF) between the images at 0 and 100 s or 0 and 15 s are shown. Scale bar, 30 μm. (**f**) Ca^2+^ elevation in individual HIT-T15 cells after 100 or 15 s of stimulation shown in **e**. The expression levels of mCherry (relative fluorescence unit, r.f.u.) and Fluo-8/AM fluorescence changes (ΔF/F) of individual HIT-T15 cells are plotted. The red lines represent the regression lines. Data are shown for *n*=144 cells (hBACCS2), *n*=133 cells (hBACCS2-i-ORAI1), and *n*=141 cells (dmBACCS2-i-dOrai). *r*, Pearson correlation coefficient. Pseudocoloured images are shown in **a**,**c**, and **e** as in Fig. 2a. Data in **b** and **d** represent means±s.e.m. The cell numbers analysed are indicated in parentheses in **b** and **d**. All data were obtained from more than three independent experiments.

To examine the applicability to various types of cells, *hBACCS2* or *dmBACCS2-IRES-dOrai* was expressed in other cell types, namely COS-7 cells (derived from African green monkey kidney), mouse cultured hippocampal neurons and HIT-T15 hamster pancreatic β-cells, and Ca^2+^ imaging was performed (Fig. 3). Efficient elevation of intracellular Ca^2+^ was observed in most COS-7 cells (Fig. 3a,b) and mouse hippocampal neurons (Fig. 3c,d) on photoactivation. The extracellular solution for the hippocampal neurons contained tetrodotoxin, a sodium channel blocker, thus excluding any effect of the action potential. In general, excitable cells such as neuronal cells express Orai proteins, but they provide little contribution to any Ca^2+^ influx^39^. Our results suggest that *hBACCS2* can be applied to both excitable and non-excitable cells. In HIT-T15 cells, a comparison of hBACCS2 expression and intracellular Ca^2+^ elevation revealed that higher expression of hBACCS2 resulted in a smaller response, while lower expression resulted in a larger response (Fig. 3e,f). In contrast, a Ca^2+^ response was observed in a large population of HIT-T15 cells expressing *hBACCS2-IRES-ORAI1* or *dmBACCS2-IRES-dOrai* regardless of the expression level (Fig. 3e,f). These results indicate that the level of endogenous Orai expression could vary substantially among HIT-T15 cells.

These findings suggest that our strategy can generate Ca^2+^ signals in most mammalian cell types under blue light illumination, and that dmBACCS2 induces faster and larger intracellular Ca^2+^ elevation compared with hBACCS2 regardless of the cell types involved.

### Spatiotemporal generation of Ca^2+^ signals

**Figure 4:**
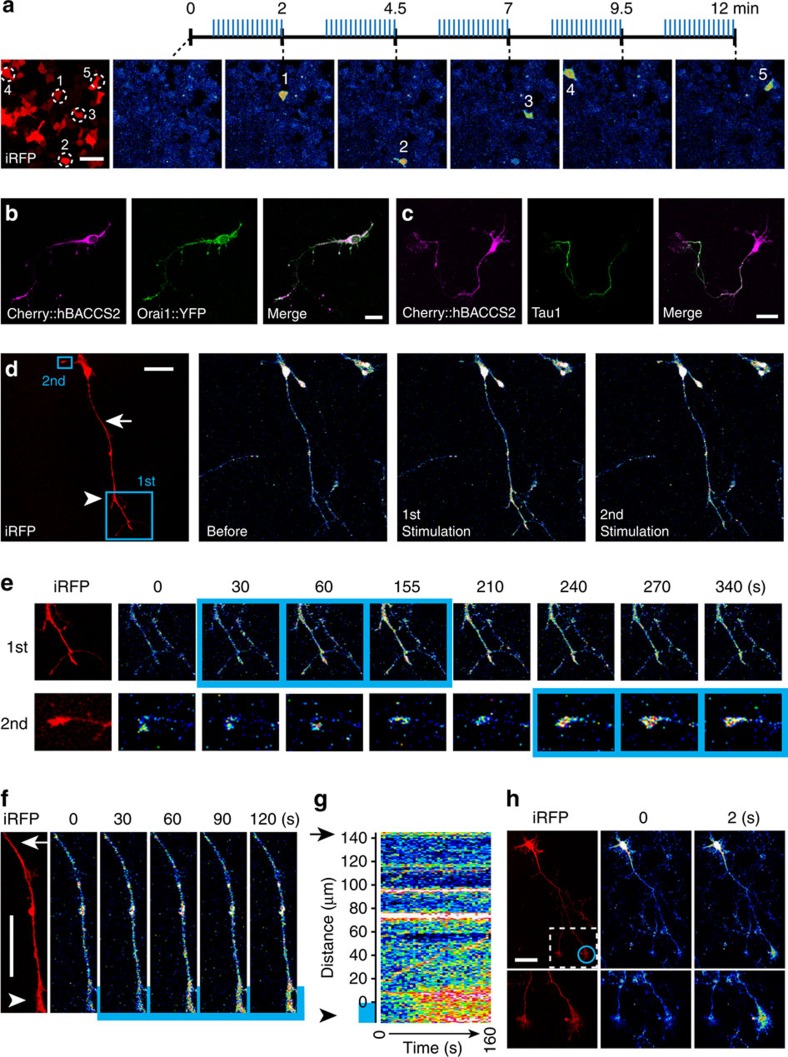
Local generation of intracellular Ca^2+^ signals. (**a**) Time series of fluorescence images of Rhod-3/AM-loaded HEK293T cells transiently expressing *hBACCS2-IRES-iRFP* during the course of photoactivation. Five target cells in circles were sequentially illuminated with a 488-nm laser (15 times; 0.2 Hz; illumination indicated by blue vertical lines). Scale bar, 50 μm. (**b**) Fluorescence images of a hippocampal neuron expressing mCherry::hBACCS2 (magenta) and ORAI1::YFP (green). Scale bar, 20 μm. (**c**) Fluorescence images of a hippocampal neuron expressing *mCherry::hBACCS2-IRES-ORAI1*. The localization of mCherry::hBACCS2 is shown in magenta and a putative axon is stained with an anti-Tau1 antibody (Alexa 488; green). Scale bar, 40 μm. (**d**) Fluorescence images of a Fluo-4/AM-loaded hippocampal neuron transiently expressing *hBACCS2-IRES-mem::iRFP*. The left panel shows the iRFP image with the positions of the first and second local activations with a 458-nm laser (blue boxes). Scale bar, 50 μm. (**e**) Higher-magnification views of the activated areas in **d**. The time points of photoactivation are indicated by blue boxes. (**f**) Higher-magnification views at the border of the first photoactivation in **d**. The time points and location of photoactivation are indicated by blue labels. Scale bar, 50 μm. (**g**) Kymograph representing the spatiotemporal activation pattern in **f**. The intensity of Fluo-4/AM fluorescence along the axon is shown as a heatmap. The positions indicated by the arrowhead and arrow correspond to the locations of the axons indicated by the same symbols in **d** and **f**. The location of the photoactivated area is indicated by the blue bar. The border of the photoactivation is set to distance 0. (**h**) Fluorescence images of a Fluo-4/AM-loaded hippocampal neuron transiently expressing *dmBACCS2-IRES-dOrai-IRES-iRFP*. The left panel shows the iRFP image with the position of the local activation with a 458-nm laser (blue circle). Higher-magnification views of the activated area (box) are shown below. Scale bar, 50 μm. Pseudocoloured images are shown in **a**,**d**,**e**,**f**,**g** and **h** as in Fig. 2a.

To examine whether Ca^2+^ signals could be generated with spatial specificity, Ca^2+^ imaging was performed in HEK293T cells expressing *hBACCS2* with focused light illumination on a specific population of cells. In this experiment, near-infrared fluorescent protein (iRFP) was used as a marker for transfected cells, thereby making it possible to image *hBACCS2*-expressing cells with simultaneous blue light stimulation and Ca^2+^ imaging. Five cells loaded with the Ca^2+^ indicator dye Rhod-3/AM were sequentially illuminated with a 488-nm laser, and intracellular Ca^2+^ elevation was specifically observed in the targeted cells (Fig. 4a). Next, mouse cultured hippocampal neurons were examined for subcellular local photoactivation. We confirmed that hBACCS2 and ORAI1 were detected throughout the neuronal structure, including the dendrites and axon, by fluorescence imaging and immunostaining (Fig. 4b,c). We then carried out Fluo-4/AM Ca^2+^ imaging of neurons expressing *hBACCS2*. The photoactivation was initially focused on the tip of the axon and then focused on a dendrite. Ca^2+^ signals were clearly elevated in the axon terminal first and then in the dendrite (Fig. 4d,e). The Ca^2+^ elevation was restricted to a small area and the signal did not show long-distance transmission (<20 μm) (Fig. 4f,g). We also performed a subcellular light illumination in hippocampal neurons expressing *dmBACCS2-IRES-dOrai*, and observed much faster local intracellular Ca^2+^ elevation than that for *hBACCS2* (Fig. 4h).

### Control of cellular events

**Figure 5:**
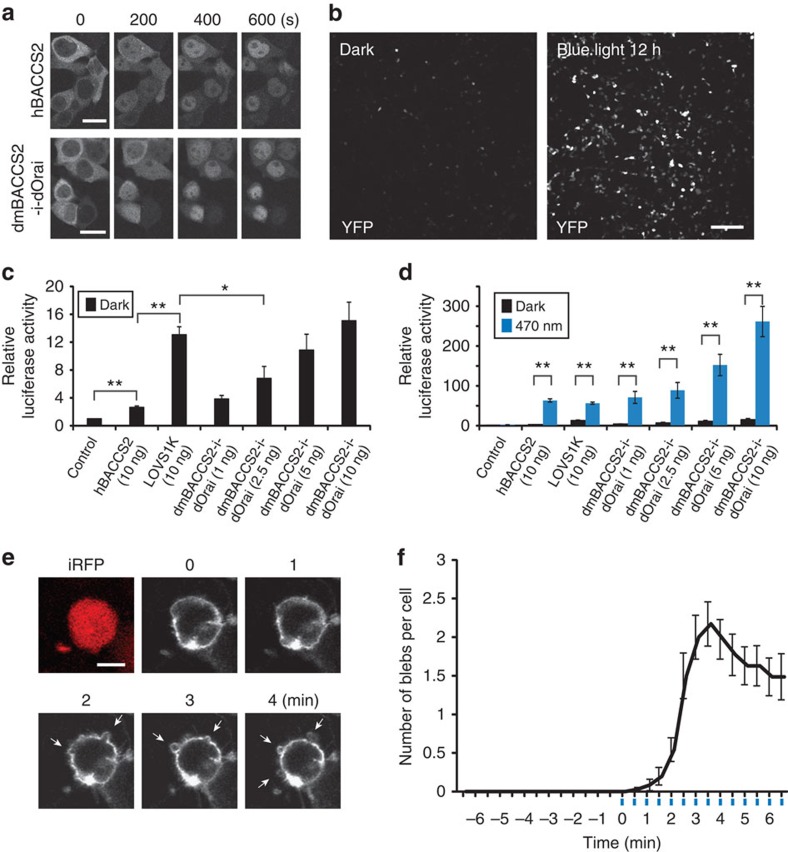
Induction of cellular responses by manipulating intracellular Ca^2+^ signals. (**a**) Time-lapse imaging of NFAT::mCherry translocation in HEK293T cells. Cells transiently expressing *hBACCS2-IRES-NFAT::mCherry* or *dmBACCS2-IRES-dOrai-IRES-NFAT::mCherry* were illuminated with a 488-nm laser every 2 min. Scale bar, 20 μm. (**b**) Light-dependent YFP upregulation in HEK293T cells transfected with *dmBACCS2-IRES-dOrai* and *P*_*NFAT*_*-YFP*. Cells were continuously illuminated with 470-nm LED blue light of 60 μW mm^−2^ or left in the dark for 12 h. Scale bar, 200 μm. (**c**,**d**) Light-dependent induction of luciferase expression after 6 h in HEK293T cells co-transfected with pGL4.30 (P_NFAT_-luc2P), pRL-SV40 (P_SV40_-hRluc, internal control,) and various Ca^2+^ photoswitch constructs. Cells were left in the dark (**c**) or continuously illuminated with 470-nm LED blue light of 60 μW mm^−2^ (**d**) for 6 h (*n*=4–6 independent experiments). Significance was assessed by *t*-test: *P<0.05, **P<0.01. (**e**) Representative images of bleb induction in HEK293 cells. Cells transiently expressing *hBACCS2-IRES-mem::iRFP* and *Lyn-CaRQ* (*YFP* fusion) were stimulated (0.1 Hz) and observed with a 488-nm laser. Scale bar, 20 μm. (**f**) Time course of bleb induction by stimulation (488-nm laser; 0.1 Hz; illumination indicated by blue vertical lines). Among cells transfected with both *hBACCS2-IRES-mem::iRFP* and *Lyn-CaRQ*, 35 of 78 cells initiated blebbing after photoactivation, compared with 0 of 83 cells transfected with *Lyn-CaRQ* alone. The numbers of blebs per cell were counted during the time course of photoactivation (*n*=35), and cells in which blebs were not induced were excluded from the analysis. Data represent means±s.e.m. in **c**,**d** and **f**.

To evaluate BACCS for cell biological applications, we first tested the light-activated switch in transcriptional regulation. NFAT is a transcription factor that regulates the expressions of cytokines, growth factors and their receptors, proteins involved in cell–cell interactions and many microRNAs^40^; elevation of intracellular Ca^2+^ induces dephosphorylation of NFAT, followed by its nuclear translocation^22^. HEK293T cells expressing *hBACCS2-IRES-NFAT::mCherry* or *dmBACCS2-IRES-dOrai-IRES-NFAT::mCherry* were stimulated with a 488-nm laser every 2 min. Live-cell imaging of NFAT::mCherry revealed that NFAT molecules, which were initially distributed in the cytoplasm, translocated to the nucleus within 10 min (Fig. 5a). To examine whether BACCS photoactivation could induce gene expression, HEK293T cells were co-transfected with *dmBACCS2-IRES-dOrai* and pHY41 (P_NFAT_-YFP-pA)^19^ encoding yellow flurescent protein (YFP) under the control of a promoter with an NFAT response element. After 12 h of blue light exposure, YFP expression was induced (Fig. 5b). To quantify the level of induction, we performed luciferase assays. *hBACCS2*, *LOVS1K* and *dmBACCS2-IRES-dOrai* were compared (Fig. 5c,d). Significant luciferase induction was observed for hBACCS2 after 6 h of light exposure. Low, but slightly elevated, basal luciferase activity in the dark was observed in cells expressing hBACCS2. This could reflect a small population of cells with an elevated basal Ca^2+^ level. LOVS1K induced luciferase activity at a similar level to that of hBACCS2 on light exposure, but showed high basal activity in the dark, consistent with a higher basal Ca^2+^ level. dmBACCS2 induced robust luciferase activity on light exposure compared with hBACCS2 and LOVS1K. dmBACCS2 also showed basal activity in the dark, which decreased with lower amounts of transfected DNA. The dynamic ranges of the induced luciferase activity for hBACCS2 and dmBACCS2 in this assay were much higher than that for LOVS1K.

We also used hBACCS2 to initiate the formation of blebs, spherical membrane protrusions, in HEK293 cells in combination with Lyn-CaRQ^41^, an engineered membrane-anchored Ca^2+^-sensitive RhoA protein that becomes activated in response to Ca^2+^ signals. Plasma membrane blebs have been implicated in apoptosis, cytokinesis and cell migration^42^. Cells expressing both *hBACCS2* and *Lyn-CaRQ* were photoactivated with a 488-nm laser. Blebs were induced in a stimulation-dependent manner (Fig. 5e), and the number of induced blebs reached the maximum level within 3.5 min (Fig. 5f).

Thus, BACCS can be applied to the control of cellular events such as NFAT-dependent transcription and cell morphological changes.

### Electrophysiological response in mouse olfactory epithelium

**Figure 6:**
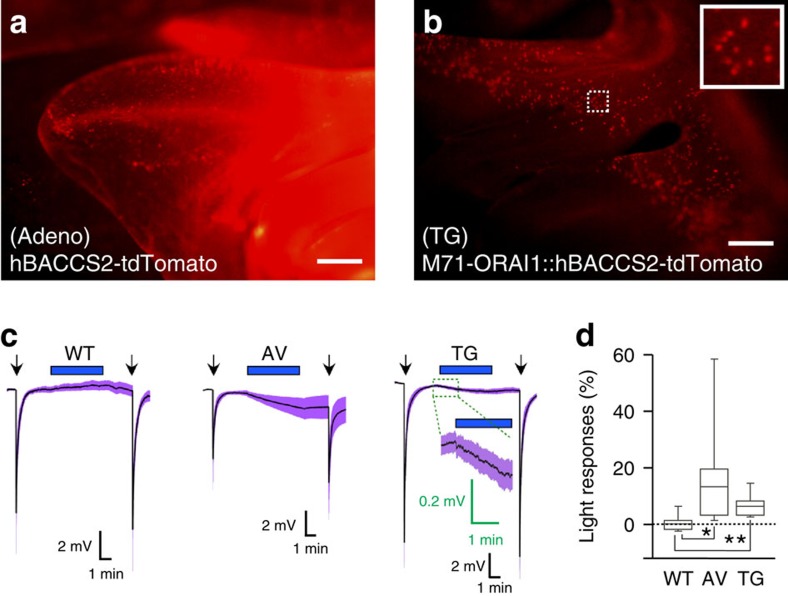
EOG analyses of odorant- and light-induced responses. (**a**) Representative fluorescence image of part of the C57BL/6 wild-type (WT) mouse olfactory epithelium infected with the adenovirus (AV) *hBACCS2-IRES-mem::tdTomato*. Scale bar, 200 μm. (**b**) Representative fluorescence image of part of the olfactory epithelium from an *M71-IRES-ORAI1::hBACCS2-IRES-mem::tdTomato* transgenic (TG) mouse. The inset shows a higher-magnification view of the olfactory epithelium. Scale bar, 200 μm. The mice in **a** and **b** were 8 weeks of age. (**c**,**d**) Light-induced depolarizing potentials recorded from *BACCS*-expressing olfactory sensory neurons. (**c**) Average EOG responses of WT mice (left), AV-infected mice (middle) and TG mice (right) to blue light illumination (blue bars). The arrows indicate the timing of the 100-ms amyl acetate application to the olfactory epithelium. Shaded regions around the EOG response traces represent the s.e.m. (**d**) Box plot of the maximal EOG responses to 4 min of blue light illumination. The *y* axis represents the EOG responses induced by blue light illumination relative to those induced by amyl acetate exposures. The data shown are for *n*=10 WT mice, *n*=16 AV-infected mice and *n*=12 TG mice. Significance was assessed by *t*-test: *P<0.05, **P<0.01.

To examine whether BACCS is applicable to mouse tissues, we generated a recombinant adenovirus for expression of *hBACCS2-IRES-mem::tdTomato*, and infected the adenovirus into the olfactory epithelium (Fig. 6a). We recorded local field potentials from the surface of the olfactory epithelium in an *ex vivo* whole-mount preparation, the electro-olfactogram (EOG)^43^. Figure 6c shows the waveforms of the average EOG responses to blue light and the odorant amyl acetate as a positive control. Significant responses to both blue light and amyl acetate were recorded at the infected surface of the olfactory epithelium on blue light exposure, while the olfactory epithelium of wild-type mice only showed responses to amyl acetate (Fig. 6c,d), suggesting that hBACCS2 was photoactivated in olfactory sensory neurons (OSNs).

To further consolidate the EOG results, we generated a transgenic mouse strain possessing a subset of OSNs that expressed *ORAI1::hBACCS2*. A DNA fragment containing the odorant receptor gene *M71* (also known as *Olfr151*) was used to generate the transgenic mouse strain^44^, in which neurons expressing the transgene *M71* co-expressed *ORAI1::hBACCS2* and *mem::tdTomato* (Supplementary Fig. 6a). The *M71* transgene was expressed in a subset of OSNs (Fig. 6b), and their axonal projection to the olfactory bulb and neural survival appeared normal (see Supplementary Fig. 6b–f for details). We carried out EOG using the transgenic mice, and observed electrophysiological responses in these mice (Fig. 6c,d). The responses in the transgenic mice were smaller, but less variable, than those in the adenovirus-infected mice, suggesting that the specific and stable expression of BACCS in transgenic animals enabled the acquisition of more stable EOG responses.

Thus, BACCS can be used in the mouse olfactory epithelium and will be applicable to *in vivo* studies.

### Fast and slow recovery kinetic mutants of dmBACCS2

**Figure 7:**
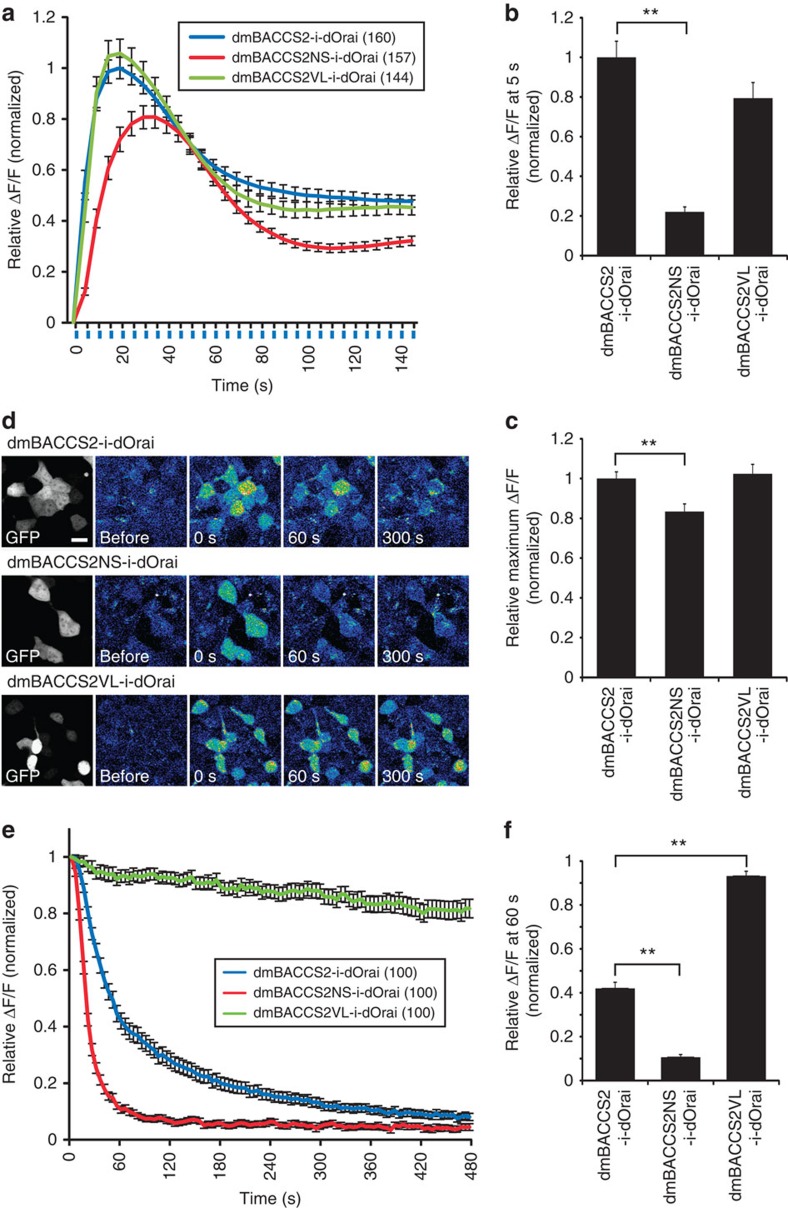
Fast and slow recovery kinetic mutants of dmBACCS2. (**a**) Time course of Fluo-8/AM fluorescence changes in HEK293T cells transiently expressing dmBACCS2 variants (dmBACCS2, dmBACCS2NS or dmBACCS2VL) with dOrai and mCherry. Cells were stimulated with a 488-nm laser (0.2 Hz; illumination indicated by blue vertical lines). All data were normalized to the mean ΔF/F for *dmBACCS2-IRES-dOrai* at 20 s for ease of comparison. (**b**) Mean Fluo-8/AM fluorescence changes at 5 s in **a**. Data were normalized to the value for *dmBACCS2-IRES-dOrai*. (**c**) Mean maximum Fluo-8/AM fluorescence changes in cells during the course of photoactivation in **a**. Data were normalized to the value for *dmBACCS2-IRES-dOrai*. (**d**) Rhod-3/AM fluorescence images of HEK293T cells transiently expressing dmBACCS2 variants with dOrai. After photostimulation with a 488-nm laser (0.2 Hz) for 170 s, cells were imaged. Pseudocoloured images are shown as in Fig. 2a. Scale bar, 20 μm. (**e**) Time course of Rhod-3/AM fluorescence decreases in HEK293T cells transiently expressing dmBACCS2 variants after photostimulation 35 times with a 488-nm laser (0.2 Hz). All data were normalized to the mean ΔF/F for each construct at 0 s. (**f**) Mean Rhod-3/AM fluorescence changes at 60 s in **e**. Data were normalized as in **e**. Significance was assessed by *t*-test: **P<0.01 in **b**,**c** and **f**. Data in **a**,**b**,**c**,**e** and **f** represent means±s.e.m. The cell numbers analysed are indicated in parentheses in **a** and **e**. All data were obtained from more than three independent experiments.

**Figure 8:**
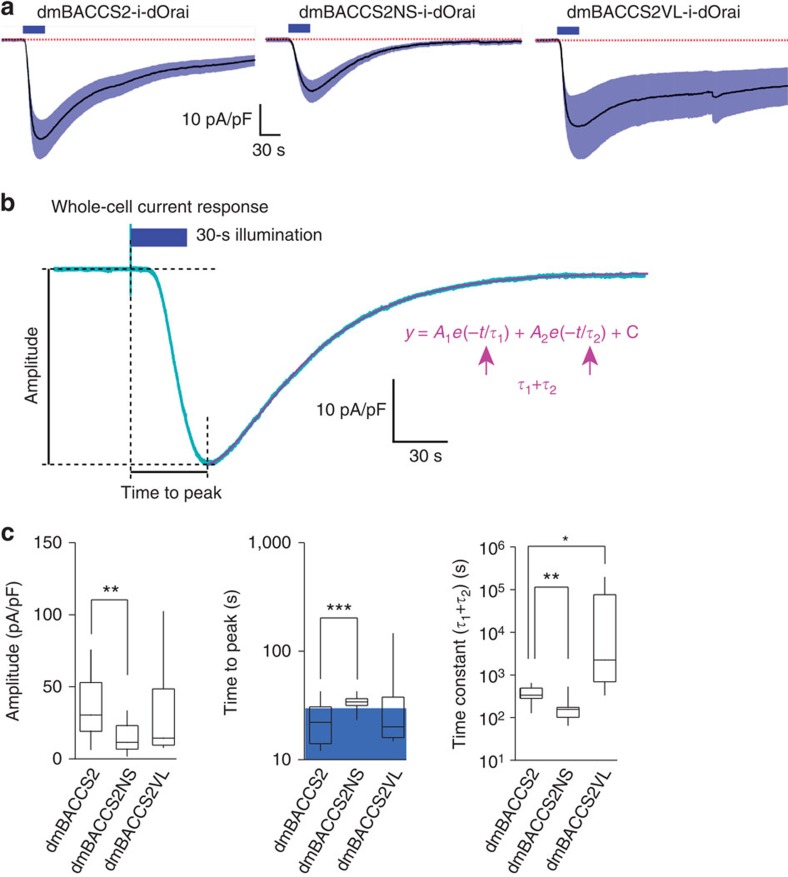
Characterization of light-activated whole-cell inward currents recorded from HEK293T cells expressing mutant *dmBACCS2-IRES-dOrai*. (**a**) Average current responses in HEK293T cells expressing mutant *dmBACCS2-IRES-dOrai* to 30 s of LED light (470 nm) illumination (blue bars): *n*=14 (dmBACCS2); *n*=12 (dmBACCS2NS); *n*=11 (dmBACCS2VL). The shaded region around the trace represents s.e.m. The dotted lines indicate the baseline. (**b**) Parameters for the current analysis. Response amplitude, time to peak (time from start of illumination to peak of response) and time constant of termination (sum of *τ*_1_ and *τ*_2_ determined by fitting the decay phase of the response to a double exponential equation; magenta) were calculated. (**c**) Box plots for a summary of the kinetic analyses of mutant dmBACCS2. The blue box indicates the timing of the illumination. Significance was assessed by *t*-test: *P<0.05, **P<0.01, ***P<0.001.

dmBACCS2 is an efficient Ca^2+^ photoswitch in mammalian cells, but still suffers from slow recovery kinetics (time constant of the whole-cell current decay phase) (Fig. 2h)^45^, rendering it difficult to precisely control its temporal signalling activities. Recently, mutational analysis of the LOV2 domain was conducted to obtain fast- and slow-cycling LOV2 variants^46^,^47^. We introduced such mutations into the LOV2 domain of dmBACCS2; N425S and V416L^46^ resulted in fast and slow switch-off phenotypes, respectively. We performed Ca^2+^ imaging in HEK293T cells expressing *dmBACCS2-IRES-dOrai*, *dmBACCS2-N425S (dmBACCS2NS)-IRES-dOrai* or *dmBACCS2-V416L (dmBACCS2VL)-IRES-dOrai*, and compared the Ca^2+^ responses. The amplitude of the Ca^2+^ increase in cells expressing the *dmBACCS2NS* construct was smaller than those in cells expressing the *dmBACCS2* or *dmBACCS2VL* constructs (Fig. 7a–c). To analyse the recovery kinetics, the intracellular Ca^2+^ decrease was observed every 5 s after 170 s of photostimulation (Fig. 7d). Notably, dmBACCS2NS had faster recovery kinetics and dmBACCS2VL had slower recovery kinetics than dmBACCS2 (Fig. 7e,f). Whole-cell patch-clamp recording revealed the precise time courses of dOrai activation and inactivation for dmBACCS2 and the two mutants (Fig. 8a). The results for three kinetic parameters, amplitude, time to peak and recovery time constant (Fig. 8b,c) were consistent with the Ca^2+^ imaging observations. The recovery time constant for dmBACCS2NS is less than one-half of that for dmBACCS2, while the time constant for dmBACCS2VL was >100 times larger than that for dmBACCS2.

Thus, the recovery kinetics of BACCS are tuneable, and both fast and slow recovery switches can be developed for specific experimental purposes.

## Discussion

We have engineered BACCS, an optogenetic tool for generating intracellular Ca^2+^ signals. Compared with previously reported Ca^2+^ optogenetic tools, some variants of BACCS were endowed with features of fast and large response induction and better off-recovery efficiency, thereby making BACCS a more useful tool for precisely generating Ca^2+^ signals.

The four BACCS variants, hBACCS1, hBACCS2, ORAI1::hBACCS2 and dmBACCS2, had different properties in their Ca^2+^ responses. Although the stoichiometry of ORAI1 and STIM1 remains controversial, Li *et al*.^28^ proposed a model in which one STIM1 dimer can interact with one ORAI1 subunit in the pore formed by tetrameric ORAI1 and octameric STIM1 to maximally open the ORAI1 channel. Consistent with this model, hBACCS2, a tandem dimer of hBACCS1, was a more efficient photoswitch than hBACCS1. A fusion protein of hBACCS2 with the C terminus of ORAI1 was also functional as a switch in HEK293T cells, but the photoactivation time course of ORAI1::hBACCS2 was slower than that of hBACCS2. Although we observed no drastic differences in Ca^2+^ responses among the three ORAI1–hBACCS2 fusion constructs with different linker lengths, the location and length of the linker might still influence the function. Alternatively, endogenous ORAI1 and ORAI1::hBACCS2 might form a heterogeneous tetramer, resulting in insufficient maximal activation of the channel. dmBACCS2 was a much more efficient photoswitch but required coexpression of dOrai in mammalian cells. We observed that the intracellular Ca^2+^ increase occurred within 1 s in HEK293T cells expressing *dmBACCS2-IRES-dOrai*, although the Ca^2+^ response was not as fast in *Drosophila* S2 cells expressing *dmBACCS2*. Mammalian cells may lack negative regulatory mechanisms for dOrai function. Although we cannot exclude the possibility that the fast Ca^2+^ response arose through functionally better stoichiometry of dmBACCS2 and dOrai in cells expressing *dmBACCS2-IRES-dOrai*, this seems unlikely because expression of *hBACCS2-IRES-ORAI1* did not result in faster or larger intracellular Ca^2+^ elevation compared with expression of *hBACCS2* alone. It has been reported that Orai1-interacting STIM1 domains, CAD^24^ and SOAR^25^, can interact with voltage-gated Ca^2+^ Ca_v_1.2 channels^48^,^49^ and Trpc channels^50^, although the domains themselves do not activate these channels. Therefore, hBACCS2, which contains a similar sequence to CAD/SOAR, may interact with such molecules, but should generate no signals through them.

We examined the basal Ca^2+^ level by two methods, comprising direct measurement by Fura-2/AM and indirect measurement by the NFAT::GFP assay. Although the Fura-2 method measures exact intracellular Ca^2+^ concentrations, we cannot exclude the possibility that BACCS could be slightly activated in the darkroom because of the difficulty in achieving complete darkness during the experiments. However, the NFAT::GFP assay would not be affected by this problem because NFAT::GFP was fixed in the darkroom before possible NFAT::GFP translocation, if any. The results for both the NFAT::GFP assay and the measurement by Fura-2/AM indicated that the mean basal Ca^2+^ levels in *hBACCS2*-expressing cells and *dmBACCS2-IRES-dOrai*-expressing cells were within the physiological range. However, BACCS-expressing cells, especially dmBACCS2-dOrai-expressing cells, contained a population of cells with a high basal Ca^2+^ level, probably because of intrinsic activity of BACCS or the difficulty in achieving complete darkness for the experiments, and these cells are likely to have influenced the results of the luciferase assay. The basal Ca^2+^ concentration could be controlled and kept at a lower level by avoiding high-level expression of the BACCS gene, at least partially, as demonstrated in the luciferase assay findings.

In what kinds of cells can BACCS be used? dmBACCS2-dOrai was successfully used in *Drosophila* S2 cells, mammalian HEK293T cells, COS-7 cells, HIT-T15 cells and hippocampal neurons, suggesting that it can be used in a wide variety of species and cell types. hBACCS2 also functioned in all mammalian cell types examined, and can be used to photoactivate endogenous Orai. The efficiency of intracellular Ca^2+^ signal elevation by light relies on the level of endogenous Orai expression in each cell. The cytosolic STIM1 C terminus has been reported to stimulate not only human ORAI1, but also ORAI2 and ORAI3 (ref. 51), indicating that hBACCS2 can activate all ORAI proteins by light illumination. On the basis of previous observations that Orai proteins are widely expressed in most mouse and human tissues^29^,^30^,^31^, hBACCS2 expression alone is likely to be sufficient for the generation of Ca^2+^ signals through endogenous Orai in most mammalian cells on light illumination. Although Stim and Orai proteins are evolutionarily conserved across metazoans^52^,^53^, direct cross-species interactions between Stim and Orai molecules appear to be inefficient between humans and *Drosophila*.

Two Ca^2+^-selective photoswitches have been reported, PACR (ref. 21) and LOVS1K (ref. 20). PACR is composed of calmodulin fused with the M13 peptide and LOV2-Jα, and releases Ca^2+^ upon light exposure. The expression can be restricted to a particular organelle, thereby allowing the control of Ca^2+^ signals at the organelle level. PACR induces Ca^2+^ concentration changes of 10–90 nM in HeLa cells, which are not sufficient to control many Ca^2+^-regulated cellular events because micromolar changes in Ca^2+^ concentration are observed in many biological responses. LOVS1K and hBACCS are both composed of the same photosensory module, LOV2-Jα, and effector domain, STIM1, with different sizes of each fragment: LOV2-Jα (404–538) and STIM1 (347–448) for hBACCS and LOV2-Jα (404–546) and STIM1 (233–450) for LOVS1K. Pham *et al*.^20^ reported that repeated photoactivation of LOVS1K co-expressed with ORAI1 gradually caused intracellular Ca^2+^ accumulation to ∼500 nM over 40 min. However, it can be estimated from our experiments that intracellular Ca^2+^ elevation to ∼500 nM can be achieved in 30 s in cells expressing LOVS1K alone (Fig. 2b,e), probably reflecting differences in the experimental setup. One non-negligible defect of LOVS1K is its high basal activity, which was observed in our NFAT::GFP assay and luciferase assay in the dark. The basal Ca^2+^ level, which affects NFAT-dependent transcription, could lead to considerable interference with cell function.

BACCS has the following features and advantages. hBACCS2 can be used to induce intracellular Ca^2+^ elevation to ∼500 nM in 30 s and >1 μM in 60 s, and its basal activity in the dark is relatively low compared with LOVS1K. hBACCS2 functions in the mouse olfactory system. In M71 transgenic mice, OSNs expressing ORAI1::hBACCS2 appeared normal in terms of cell survival and axonal projection^54^, suggesting that the basal Ca^2+^ concentration induced by ORAI1::hBACCS2 was sufficiently low to have no effect on the development of olfactory neurons. The relatively low basal activity and high Ca^2+^ response (micromolar change) make hBACCS2 suitable for application to many Ca^2+^-dependent biological events both *in vitro* and *in vivo*. dmBACCS2-dOrai is by far the most efficient and robust Ca^2+^ photoswitch in mammalian cells. Although basal activity in the dark can be observed when dmBACCS2-dOrai is expressed at a high level, the dynamic range of the Ca^2+^ response is wide, and fast Ca^2+^ intracellular elevation can be induced. We observed that both its magnitude and speed of Ca^2+^ elevation were better in all types of tested cells expressing *dmBACCS2-IRES-dOrai* than in cells expressing *hBACCS2*, suggesting that dmBACCS2-dOrai is a more useful tool to precisely induce Ca^2+^ responses. Furthermore, as dmBACCS2 does not appear to interact with human ORAI, the dmBACCS2-dOrai system is likely to function without interacting with other endogenous molecules in mammalian cells. Therefore, hBACCS2 and dmBACCS2 are alternative candidates for practical use in mammalian cells (Supplementary Fig. 7). hBACCS2 will be an appropriate tool to control the activity of endogenous Orai and Orai-mediated cellular events, such as induction of T-cell activation^22^, turning of growth cones^55^ and cell migration^56^. The dmBACCS2-dOrai system may be used with better temporal resolution for controlling general Ca^2+^-dependent cellular events without interfering with the functions of other endogenous molecules.

Although the activation time was improved by using dmBACCS2, the recovery kinetics would be a limiting factor for inter-trial frequency. In cases of synaptic transmission or cardiac contraction, the Ca^2+^ effector systems respond to pulses within the microsecond to millisecond range^9^. The photocycles of PACR, LOVS1K, hBACCS2 and dmBACCS2 all depend on LOV2-Jα and their recovery kinetics will be similar; LOV2-Jα recovers spontaneously with half-times of ∼50 s (ref. 45). As an effort to tune the recovery kinetics, we introduced mutations implicated in the photocycle of LOV2-Jα (refs 46, 47) into dmBACCS2 to obtain various recovery kinetic mutants (Supplementary Fig. 7). The fast recovery mutant showed significantly faster Ca^2+^ recovery in the dark than wild-type dmBACCS2. This improvement will help substantially towards the manipulation of cellular events controlled by oscillations of Ca^2+^ transients on the second to minute timescale, including growth-cone migration/turning, axonal growth, cell migration, muscle development and release of cytokines^9^, although this mutant still falls short for the control of synaptic transmission or cardiac contraction. Further mutagenesis in the LOV2-Jα domain will produce improved dmBACCS2 with faster reversibility. The slow recovery kinetic mutant showed prolonged intracellular Ca^2+^ elevation after the lights were turned off. It will require less light illumination for sustained activation of the photoswitch, which could avoid undesirable phototoxicity to cells and unfocused activation through drifting of the target sample. The fact that the introduction of the mutations for the fast- and slow-cycling LOV2-Jα into dmBACCS2 resulted in fast and slow recovery kinetics of dmBACCS2-mediated dOrai responses, respectively, is consistent with the idea that these mutations changed the LOV2-Jα kinetics, resulting in the tuning of the binding of dmBACCS2 to dOrai.

One of the advantageous features of photoactivatable molecules is the ability to control their activity with high spatial resolution. By focused illumination of a blue light laser, we successfully activated local Ca^2+^ signals at axon terminals and dendrites of hippocampal neurons. Ca^2+^ microdomains are sites with localized high Ca^2+^ concentrations near Ca^2+^ channels at the cell surface or internal stores^11^. Ca^2+^ microdomains enable different subcellular domains to execute many distinct cellular processes with specificity in single cells. For example, Ca^2+^ microdomains in spiny/aspiny dendrites, soma, mossy fibres and synaptic endings are responsible for distinct neuronal functions of learning and memory, gene transcription and transmitter release^11^. Thus, light manipulation of distinct Ca^2+^ microdomains in single cells will contribute to the elucidation of subcellular events with high spatial resolution.

The ability of BACCS to generate global and subcellular Ca^2+^ signals, and our application of BACCS to various cell types and transgenic animals suggest that BACCS will be a useful optogenetic tool for generating intracellular Ca^2+^ signals both *in vitro* and *in vivo*. Precise and non-invasive exploration of intracellular Ca^2+^ functions at the subcellular level by light illumination will contribute towards a deeper understanding of the numerous Ca^2+^-dependent physiological events.

## Methods

### DNA constructs

HA-NFAT1 (1–460)-GFP, human STIM1-CFP and Orai1-YFP were kind gifts from Anjana Rao (Addgene plasmids #11107, #19755 and #19756); pLyn-CaRQ and LOVS1K from Kevin Truong (Addgene plasmids #34559 and #31981); M71-TV from Peter Mombaerts (Addgene plasmid #15635); pTriEx-mVenus-PA-Rac1, pTriEx-mVenus-PA-Rac1-C450A and pTriEx-PA-Rac1-I539E from Kraus Hahn (Addgene plasmids #22007, #22021 and #22026); *Mem::tdTomato* (tdTomato with a membrane localization signal) from Kazunari Miyamichi, *iRFP*^57^ from Michiyuki Matsuda; and P_NFAT_-YFP (pHY41) from Martin Fussenegger. For details of each construct used in this study, please see the Supplementary Methods. Plasmids and sequence information for *hBACCS2*, *ORAI1::hBACCS2*, *dmBACCS2-IRES-dOrai*, *dmBACCS2NS-IRES-dOrai*, *dmBACCS2VL-IRES-dOrai* and *mRFP::dmBACCS2* will soon be available from Addgene (http://www.addgene.org).

### Cell culture and transient transfection

Cells were maintained in the following media: for HEK293T (obtained from TaKaRa), HEK293 (RIKEN Cell Bank) and COS-7 cells^58^, minimal essential medium (MEM; Nacalai Tesque) supplemented with 10% (v v^−1^) fetal bovine serum (FBS); for HIT-T15 cells (DS Pharma Biomedical Co. Ltd.), F-12K medium (WAKO Chemical Co.) supplemented with 10% (v v^−1^) horse serum and 2.5% (v v^−1^) FBS; for Jurkat cells (RIKEN Cell Bank), RPMI1640 (Nacalai Tesque) supplemented with 10% (v v^−1^) FBS; for *Drosophila* S2 cells (RIKEN Cell Bank), Schneider's medium (Life Technologies) containing 10% (v v^−1^) FBS. Two days before experiments, HEK293T, HEK293 and COS-7 cells were seeded on glass-bottomed dishes (Greiner) coated with collagen (Cellmatrix Type IC; Nitta Gelatin Inc.), and HIT-T15 and S2 cells were seeded on non-coated glass-bottomed dishes. On the next day, plasmid transfection was performed using Lipofectamine 2000 (Invitrogen) according to the manufacturer's instructions. The cells were analysed 24–36 h after transfection. Jurkat cells were seeded and transfected with plasmids using Lipofectamine 2000 according to the manufacturer's instructions. After 4 h, phorbol 12-myristate 13-acetate (PMA; Wako Chemical Co.), a specific activator of protein kinase C, was added (final concentration: 50 ng ml^−1^) to enhance the cytomegalovirus promoter activity and gene expression in Jurkat cells. The cells were analysed 8–12 h after transfection.

Primary hippocampal neurons derived from mouse embryos were cultured in accordance with the method of Kaech and Banker^59^ with some modifications^60^. Briefly, neurons were subjected to plasmid transfection using Lipofectamine 2000 after 3 or 6 days of culture for Ca^2+^ imaging and immunohistochemistry, respectively. After 2 further days, the transfected neurons were analysed.

### Immunostaining

*mCherry::hBACCS2* was transfected into hippocampal cells 6 days after dissection. The cells were fixed with 4% paraformaldehyde/PBS for 20 min, permeabilized with 0.1% Triton X-100/PBS for 15 min and incubated in 5% bovine serum albumin (BSA)/PBS for 15 min. The cells were then incubated with a mouse anti-Tau1 monoclonal antibody (PC1C6; Millipore; 1:4,000 dilution) in 2% BSA/PBS for 1 h. After blocking with 5% BSA/PBS for 15 min, the cells were incubated with an Alexa Fluor 488-conjugated goat anti-mouse IgG antibody (Invitrogen; 1:500 dilution) in 2% BSA/PBS for 30 min. Images were obtained using an LSM510 META laser scanning microscope (Carl Zeiss).

### Ca^2+^ imaging

Cells were loaded with Fluo-4/AM, Fluo-8/AM (2.5 μM for both; Invitrogen) or Rhod-3/AM (10 μM; Invitrogen) according to the manufacturer's instructions. Fluo-4/Fluo-8 and Rhod-3 were used for different wavelengths, and there were no specific reasons underlying the use of Fluo-4 and Fluo-8 for individual experiments. Lasers with wavelengths of 488 and 458 nm were used for photostimulation depending on the apparatus. The standard extracellular Ringer's solution contained 138 mM NaCl, 5.6 mM KCl, 2 mM CaCl_2_, 2 mM MgCl_2_, 9.4 mM D-glucose, 5 mM HEPES and 2 mM sodium pyruvate (pH 7.4, adjusted with NaOH) for mammalian cells, and 150 mM NaCl, 5 mM KCl, 2 mM CaCl_2_, 4 mM MgCl_2_ and 10 mM HEPES (pH 7.2, adjusted with NaOH) for S2 cells. Intracellular Ca^2+^ was monitored using an MRC-1024 confocal laser scanning microscope (Bio-Rad Laboratories) on an Axiovert 100 inverted microscope (Carl Zeiss) equipped with a × 40 C-Apochromat objective lens (Carl Zeiss), a LSM510 META laser scanning microscope on an Axiovert 200 M microscope (Carl Zeiss) equipped with a × 40 × C-Apochromat objective lens (Carl Zeiss) or an FV1200 laser scanning microscope (Olympus) on an IX83 microscope (Olympus) equipped with a × 40 × UPlanSApo objective lens. A laser line (488 nm) was used to photoactivate BACCS and image Fluo-4/AM and Fluo-8/AM in all cell types, except for local photoactivation of hippocampal neurons. Two laser lines (488 and 559 nm) were used to photoactivate BACCS and image Rhod-3/AM, respectively. Photostimulation was performed at a scan rate of 10 μs per pixel (pixel size: 0.615 μm per pixel) with a laser line (488 nm; 10%) for Fluo-8/AM and Fluo-4/AM imaging (0.2 Hz), a scan rate of 8 μs per pixel (pixel size: 1.230 μm per pixel) with a laser line (488 nm; 20%) for Fluo-8/AM imaging (10 Hz) or a scan rate of 20 μs per pixel (pixel size: 0.615 μm per pixel) with a laser line (488 nm; 20%) for Fluo-8 imaging of reversible and repeated photoactivation experiments. For light dose-response analyses, a custom-made LED (470 nm; OSUB5111A-ST; SEMITEC) and laser line (568 nm) were used to photoactivate BACCS and image Rhod-3/AM, respectively. For Ca^2+^ imaging of hippocampal neurons, tetrodotoxin was added to the extracellular solution to exclude any effect of the action potential. For local photoactivation of hippocampal neurons, Fluo-4/AM was loaded according to the manufacturer's instructions. Laser light (458 nm; 10%; 0.2 Hz) was used to photoactivate BACCS at a scan rate of 2.56 μs per pixel (pixel size: 0.64 μm per pixel; iterations: 3), and 633-nm and 514-nm laser lines were used to image iRFP and Fluo-4/AM, respectively. It should be noted that BACCS was not activated with a 514-nm laser. Image analyses were performed using ImageJ software (NIH). In all experiments, a positive Ca^2+^ response was defined as a stimulus-dependent deviation in Ca^2+^ fluorescence that exceeded twice the s.d. of the mean baseline fluorescence noise. All Ca^2+^ imaging experiments were performed at room temperature.

The number of experimental replicates is mentioned in each figure or figure legend except for the following Ca^2+^ imaging experiments: local activation of mouse hippocampal neurons expressing *hBACCS2-IRES-mem::iRFP* (*n*=27 cells from six independent experiments) or *dmBACCS2-IRES-dOrai-IRES-iRFP* (*n*=13 cells from five independent experiments).

No data points were excluded, except for cells with high Ca^2+^ concentrations before photoactivation, which were excluded from all analyses (Supplementary Table 1) and cells beyond the ranges of the Ca^2+^ indicator dyes after photoactivation, which were excluded from data analyses. For the generation of time-course curves of fluorescence changes (Figs 2b,g, 3b,d and 7a,e; Supplementary Figs 2b–f and 4c), cells that showed no Ca^2+^ response were excluded from the analyses (Supplementary Table 1), except for data in the absence of extracellular Ca^2+^, data for dmBACCS2 in Fig. 2b, and data for the control experiments in Figs 2g and 3d.

In most of the Ca^2+^ imaging experiments, an apparent decrease in ΔF/F was observed during the first few seconds, probably through bleaching of the Ca^2+^ indicators.

### Live-cell imaging and photoactivation

Time-lapse images of NFAT translocation were obtained using the FV1200 laser scanning microscope. *hBACCS2-IRES-NFAT::mCherry* or *dmBACCS2-IRES-dOrai-IRES-NFAT::mCherry* was transfected into HEK293T cells. The cells were photoactivated every 2 min at a scan rate of 20 μs per pixel (pixel size: 0.307 μm per pixel) with a laser line (488 nm; 20%) for NFAT translocation, and the NFAT::mCherry localization was imaged using the 568-nm laser every 2 min (*n*=3 independent experiments for *hBACCS2-IRES-NFAT::mCherry*; *n*=7 independent experiments for *dmBACCS2-IRES-dOrai-IRES-NFAT::mCherry*). Time-lapse images of bleb formation were obtained using the LSM510 META laser scanning microscope. *Lyn-CaRQ* (containing *YFP*) and *hBACCS2-IRES-iRFP* were co-transfected into HEK293 cells. *hBACCS2*-transfected cells were verified by *iRFP* expression. The cells with YFP fluorescence were first imaged using the 514-nm laser (no photoactivation), and then photoactivated and imaged with the 488-nm laser. Cells that showed bleb formation before photoactivation were excluded from the analyses for both *Lyn-CaRQ*/*hBACCS2-IRES-iRFP* double-transfected cells and *Lyn-CaRQ* single-transfected cells (control). These experiments were performed at room temperature.

### NFAT translocation assay

For screening of BACCS constructs, *NFAT::CFP* and *tdTomato::LOV::STIM1* were co-transfected into HEK293T cells. Cells were either illuminated with a 470-nm LED light (cat. no. NF-1050-B; NIHON EIDO) or not illuminated, for 20 min in a darkroom. The cells were then fixed with 4% paraformaldehyde.

For analyses of the basal NFAT location, *NFAT::GFP*, *hBACCS2-IRES-NFAT::GFP*, *dmBACCS2-IRES-NFAT::GFP* or *LOVS1K-IRES-NFAT::GFP* were transfected into HEK293T cells, and the cells were fixed with 4% paraformaldehyde in the darkroom 24 h after transfection. The cells were counterstained with 4,6-diamidino-2-phenylindole (Nacalai Tesque) to visualize the nuclei. Images were obtained using the LSM510 META laser scanning microscope. Cells with brighter NFAT::CFP (or GFP) fluorescence in the nucleus compared with that in the cytoplasm were defined as cells with nuclear NFAT.

### Luciferase assay and YFP induction

For the luciferase assay, HEK293T cells in a 96-well format were transfected with 50 ng per well P_NFAT_-luc2P (pGL4.30[luc2P/NFAT RE/]; Promega), 50 ng per well P_SV40_-hRluc (pRL-SV40; Promega) and 0–10 ng per well various Ca^2+^ photoswitch constructs, and incubated for 23 h. hRluc (*Renilla* luciferase) activity was used as an internal control for cell viability and transfection efficiency. After the medium was replaced with MEM supplemented with 10% FBS containing 10 ng ml^−1^ PMA, the cells were either illuminated with a 470-nm LED light (60 μW mm^−2^) or not illuminated, for 6 h in CO_2_ incubators. The firefly and *Renilla* luciferase activities were quantified using a Dual-Glo Luciferase assay system (Promega). Luminescence was measured using an ARVO X5 (PerkinElmer). For YFP induction, HEK293T cells in a glass-bottomed 35-mm dish with four compartments (Greiner Bio-One) were transfected with 1 μg per well pHY41 and 500 ng per well *dmBACCS2-IRES-dOrai*, and incubated for 24 h. The medium was then replaced with MEM supplemented with 10% FBS containing 10 ng ml^−1^ PMA, and the cells were either illuminated with a 470-nm LED light (60 μW mm^−2^) for 12 h or not illuminated, in CO_2_ incubators. YFP expression was observed using the FV1200 laser scanning microscope.

### Recombinant adenovirus infection

The recombinant cosmid vector was packaged with Gigapack III Plus Packaging Extract (Agilent) according to the manufacturer's instructions and amplified. The vector was digested with *Bsp*T1041, purified, and transfected into HEK293 cells using a CalPhos Transfection Kit (Clontech). The amplified recombinant adenovirus was used for *in vivo* infection. A total of 6–12 μl of the adenovirus solution was injected by pipetting into a mouse nostril.

### Animals

To generate *M71-IRES-ORAI1::hBACCS2-IRES-mem::tdTomato*, the transgene sequence was excised by *Pme*I digestion, purified and microinjected into the pronuclei of C57BL/6 mice. The transgenic mice were maintained in a C57BL/6 background or crossed with the *M71-IRES-tauGFP* mouse strain (a kind gift from Peter Mombaerts) to generate a transgenic mouse strain with homozygous *M71-IRES-tauGFP*. Mice of either sex were used. All animal experiments were approved by the Institutional Animal Care and Use Committee of Tokyo Medical and Dental University.

### Whole-mount imaging and cell counting

The surface of the olfactory epithelium from *M71* transgenic mice with homozygous *M71-IRES-tauGFP* at 2- and 8-week old was observed in a whole-mount preparation using the FV1200 laser scanning microscope on the IX83 microscope. The numbers of cells labelled with intrinsic GFP and tdTomato fluorescence on ectoturbinates II, II′ and III were counted. The mean counts between the left and right epithelia were used as the M71 cell numbers for each mouse. Whole-mount imaging of the olfactory bulb was performed using the LSM510 META laser scanning microscope on the Axiovert 200 M microscope (*n*=4 mice).

### Electrophysiology

For whole-cell patch-clamp recording in HEK293T cells, BACCS expression vectors were transiently transfected using the FuGENE HD Transfection Reagent (Promega). Whole-cell currents were amplified with a patch-clamp amplifier (Axopatch 200B; Molecular Devices), and then digitized with PowerLab (AD Instruments). The extracellular solutions contained 140 mM NaCl, 5 mM CsCl, 10 mM HEPES, 10 mM CaCl_2_, 1.2 mM MgCl_2_ and 30 mM D-glucose (pH 7.4). The electrode solution contained 140 mM CsCl, 10 mM NaCl, 10 mM HEPES, 2.84 mM CaCl_2_, 2.47 mM MgCl_2_ and 5 mM EGTA-2K (pH 7.4). The calculated free Ca^2+^ concentration in this solution was 100 nM. The data were low-pass filtered at 2 kHz and sampled at 10 kHz. Kinetic analysis of patch-clamp data was performed in a blinded fashion.

For perforated patch recording in Jurkat cells, cells transiently transfected with *hBACCS2-IRES-mCherry* were suspended in extracellular buffer solution containing 130 mM Na-methanesulfonate, 10 mM HEPES, 10 mM Ca(OH)_2_, 1 mM MgCl_2_ and 10 mM D-glucose (pH 7.4), and loaded on Cell-TAK-coated glass-bottomed dishes. Micropipettes of 5–10 MΩ were manufactured from borosilicate glass using a puller (PC-10; Narishige). The electrode solution contained 140 mM CsCl, 10 mM NaCl, 10 mM HEPES, 2.84 mM CaCl_2_, 2.47 mM MgCl_2_ and 5 mM EGTA-2Cs (pH 7.4). Nystatin was dissolved in methanol containing 100 mM *N*-methyl-D-glucamine, 100 mM methanesulfonic acid and 10 mM phenol red to a concentration of 5 mg ml^−1^. Subsequently, 50 μl of this nystatin solution was evaporated, and the precipitate was dissolved in 1 ml of electrode solution. The solution was loaded into the electrode through a 0.22-μm syringe filter. After GΩ seal formation by manual suction, 10–15 min was allowed for stable perforation.

For EOG recording, mice at 5–7 weeks of age were anaesthetized with diethyl ether and euthanized by decapitation. The heads of the mice were split along the midline with a scalpel, and the nasal septum was removed so that the olfactory turbinate could be exposed. The head was placed on a recording agar chamber. Slow transepithelial potential changes were recorded from the olfactory epithelium using a glass pipette (0.1–0.5-MΩ tip resistance; filled with 140 mM NaCl, 5.6 mM KCl, 5 mM HEPES, 2 mM pyruvic acid sodium salt, 1.25 mM KH_2_PO_4_, 2 mM CaCl_2_, 2 mM MgCl_2_, 9.4 mM D-glucose and 2 wt% agar, adjusted to pH 7.4), which was connected by an Ag/AgCl wire to a DC-differential amplifier (DP-301; Warner). A second Ag/AgCl wire was connected to another glass pipette inserted into the agar chamber and served as a reference electrode. Electrical signals were band-passed at DC-100 Hz, stored on an Apple computer using a 1-kHz sampling recorder, and processed with a 30-Hz low-pass digital filter. Odorant solutions were prepared in 25 ml of water as 0.1 wt% solutions. Vapour-phase odorant stimuli were generated by collection of the headspace above 25 ml of odorant solution in a sealed 100-ml glass bottle. Vapour stimuli were injected into a stream of air under the control of a combination of custom-made transistor–transistor logic-controlled electric valves (Cole-Parmer). hBACCS2 was photoactivated with an LED and a 438±24-nm band filter (SPECTRA light engine; Lumencor). The timing of the light stimulation was controlled by a custom-made transistor–transistor logic signal controller.

### Measurement of intracellular Ca^2+^ concentration

Transfected HEK293T cells were loaded with 2.5 μM Fura-2/AM for 30 min. Fluorescence was measured with an Aquacosmos Ca^2+^ imaging system and a CCD camera (ORCA-ER; Hamamatsu Photonics). Fura-2/AM was excited by a Xenon lamp (Lambda DG-4 PLUS; Sutter Instruments). For calibration of the intracellular Ca^2+^ concentration after completion of the experiments, ionomycin Ca^2+^ salt (5 μM) and the Ca^2+^ chelator EGTA (10 mM) were added to chelate the Ca^2+^ in the chamber and obtain the minimal Fura-2 fluorescence. CaCl_2_ (10 mM) was then added to the imaging chamber to obtain the maximal Fura-2 fluorescence.

The intracellular free Ca^2+^ concentration was calculated from the Fura-2 fluorescence using the following equation:


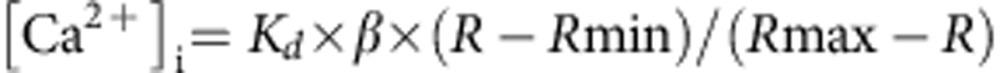


where *K*_*d*_ is the dissociation constant of the calcium complex, *β* is the minimum/maximum fluorescence ratio of Fura-2 excited at the reference wavelength of 380 nm, and *R* is the fluorescence ratio of 340/380 nm.

### Statistical analysis

Statistical analyses were performed using Excel software (Microsoft) for cell counts (*t*-test) and HIT-T15 cell analyses (Pearson correlation coefficient), and StatView (Hulinks) for EOG and whole-cell current analyses (*t*-test) and measurements of intracellular Ca^2+^ concentrations (one-way analysis of variance and *post hoc* Fisher's Protected Least Significant Difference (PLSD) test). All tests were two sided.

## Additional information

**How to cite this article:** Ishii, T. *et al*. Light generation of intracellular Ca^2+^ signals by a genetically encoded protein BACCS. *Nat. Commun.* 6:8021 doi: 10.1038/ncomms9021 (2015).

## Supplementary Material

Supplementary Information Supplementary Figures 1-7, Supplementary Table 1, Supplementary Methods and Supplementary References
