# Supplementary material for: Light generation of intracellular Ca2+ signals by a genetically encoded protein BACCS
Source: Nat Commun. 2015 Aug 18;6:8021. doi: 10.1038/ncomms9021 (PMC4557345; doi:10.1038/ncomms9021)
Supplement: Supplementary Information — Supplementary Figures 1-7, Supplementary Table 1, Supplementary Methods and Supplementary References [file ncomms9021-s1.pdf]

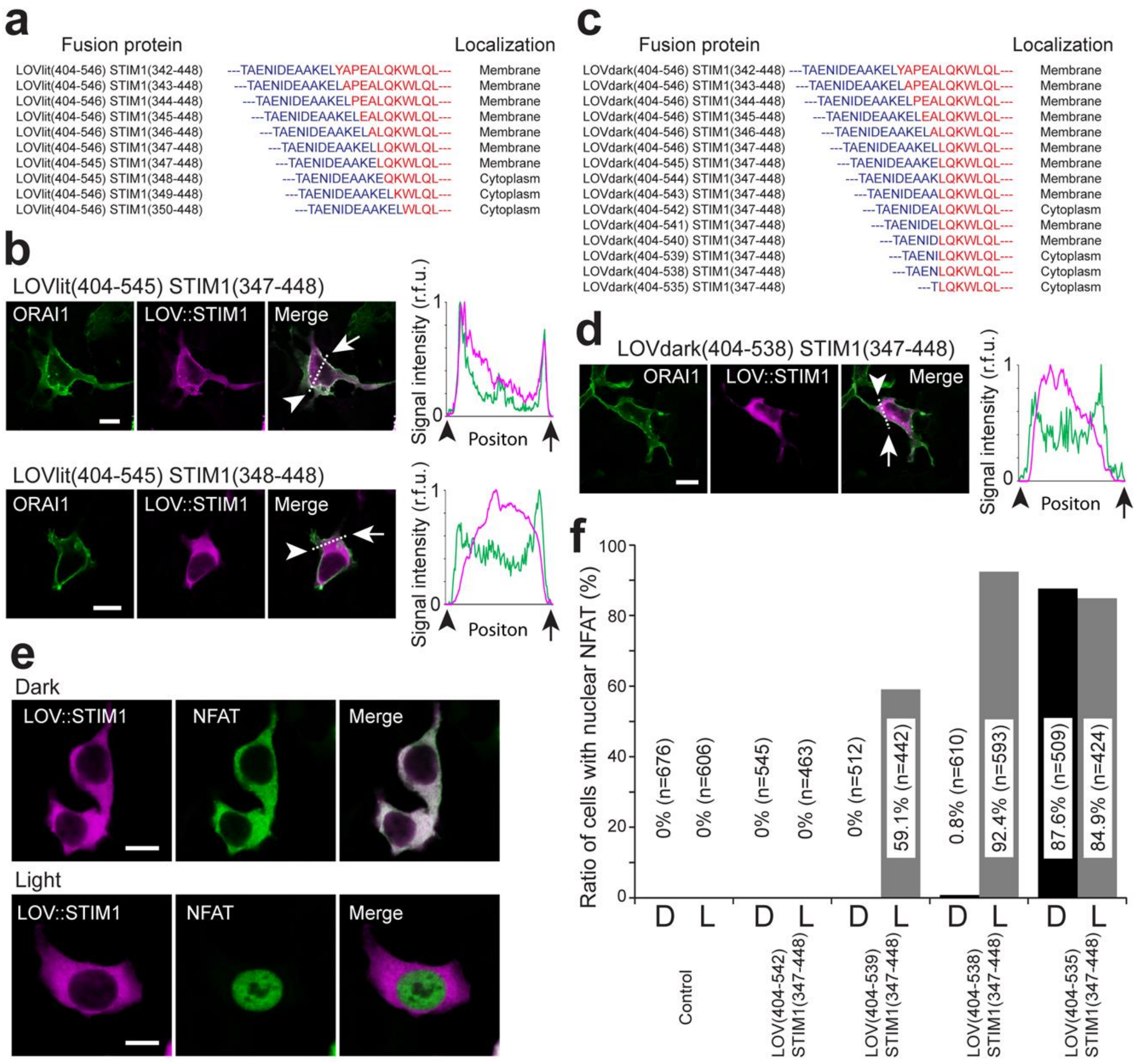

Supplementary Figure 1 | Screening of functional BACCS in HEK293T cells. (**a, b**) Screening for the minimal STIM1 fragment required for binding to ORAI1. (**a**) The lit-state mutant of the LOV2 domain (amino acids 404–546; I539E), which mimics the photoactivated form of LOV2-Jα[1], was fused to various positions at the N-terminus of STIM1 fragments (amino acids 342–448 to 350–448). The minimal STIM1 fragment (amino acids 347–448) that interacted with ORAI1 in HEK293T cells was determined. Blue, LOV2 fragment; red, STIM1. (**b**) Representative images of cells transiently expressing ORAI1::YFP (green) and tdTomato::LOV::STIM1 fusion (magenta). tdTomato::LOVlit (404-545)::STIM1 (347-448) localizes to the plasma membrane (top), whereas tdTomato::LOVlit (404-545)::STIM1 (348-448) does not (bottom). Fluorescence intensity (relative fluorescence units; r.f.u.) plots across the cells (dotted lines) are shown in the rightmost panels. The positions indicated by the arrowheads and arrows correspond to the locations indicated by the same symbols in the images. STIM1 (347-448) is the smallest N-terminal fragment effective for interaction with

ORAI1. Scale bar, 10 μm. (**c, d**) Screening of the LOV2 domain for interruption of STIM1 binding to ORAI1. (**c**) The dark-state mutant of the LOV2 domain (amino acids 404–546; C450A), which mimics the inactive form of LOV2-Jα[2], with various C-terminal ends was fused to various STIM1 fragments. Four fusion proteins that did not interact with ORAI1 in HEK293T cells were selected then further screened as shown in panels (e) and (f). Blue, LOV2 fragments; red, STIM1 fragments. (**d**) Representative images of cells transiently expressing ORAI1::YFP (green) and tdTomato::LOV::STIM1 fusion (magenta). tdTomato::LOVdark (404-538)::STIM1 (347-448) does not localize to the plasma membrane. Fluorescence intensity plots are shown as described for (b). Scale bar, 10 μm. (**e, f**) Screening for light-activatable LOV2-STIM fusion proteins. (**e**) Representative images of cells transiently expressing tdTomato::LOV (404-538)::STIM1 (347-448) (magenta) and NFAT::CFP (green). Cells were not exposed to light (top), or were exposed to 470-nm LED light for 20 min (bottom). Scale bar, 10 μm. Note that a Ca^{2+} increase in the cells induces translocation of NFAT protein from the cytoplasm to the nucleus. (**f**) Ratios of cells with NFAT nuclear localization in cells transiently coexpressing tdTomato::LOV::STIM1 fusion protein, ORAI1, and NFAT::CFP. Cells were left in the dark for 20 min or exposed to 470-nm LED light for 20 min. The ratios of cells with nuclear NFAT among transfected cells were calculated as percentages. The fusion proteins that induced translocation of NFAT from the cytoplasm to the nucleus upon blue light exposure were selected as candidate photoswitches for ORAI1. The exact values and sample sizes (n) are shown in the figure. Cells coexpressing only *ORAI1* and *NFAT* were used as controls. Data were obtained from three independent experiments. L, light; D, dark.

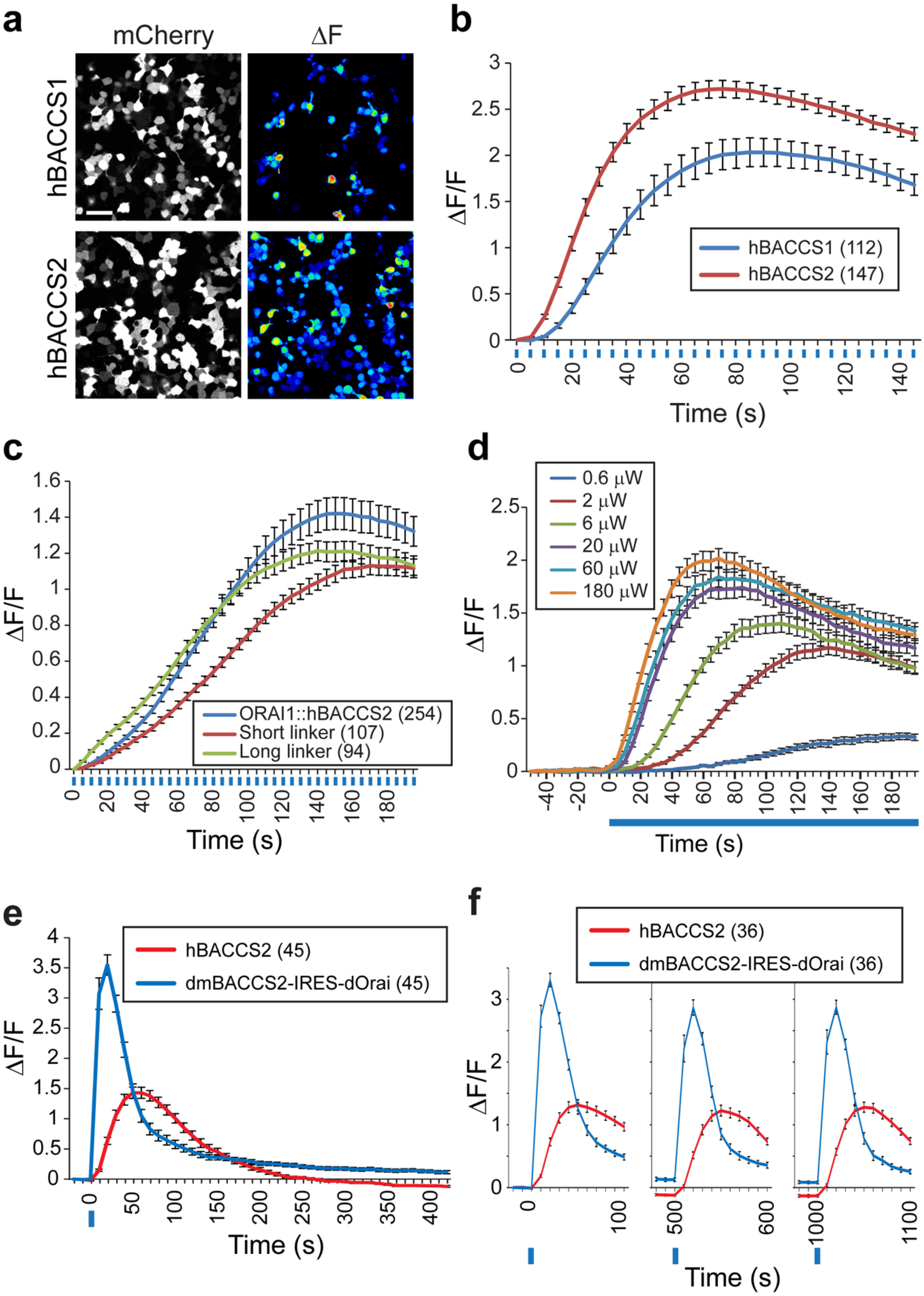

Supplementary Figure 2 | Characterization of BACCSs in HEK293T cells. (**a**) Efficient intracellular Ca^{2+} increase in cells expressing *hBACCS2* compared with those expressing

hBACCS1 upon photoactivation. Representative fluorescence images of mCherry and Fluo-8/AM fluorescence changes (ΔF) during 150 s of photoactivation are shown for cells expressing *hBACCS1-IRES-mCherry* or *hBACCS2-IRES-mCherry*. Pseudocoloured images are shown as in Fig. 2a. Scale bar, 50 μm. (**b**) Time course of Fluo-8/AM fluorescence changes in cells expressing *hBACCS1* or *hBACCS2* during the course of photoactivation (488-nm laser; 0.2 Hz; illumination indicated by blue vertical lines). (**c**) Time course of Fluo-8/AM fluorescence changes in cells expressing *ORAI1::hBACCS2* or its variants in linker length during the course of photoactivation (488-nm laser; 0.2 Hz; blue vertical lines). (**d**) Light dose-response profiles of hBACCS2 activation using the Ca^{2+} indicator dye Rhod-3/AM. Cells were continuously illuminated with 470-nm LED blue light from 0.6–180 μW mm^{-2}, as shown by the blue bar below the plot (*n*=150 cells from three independent experiments). (**e**) Reversible Ca^{2+} response after photoactivation. The time courses of Rhod-3/AM fluorescence changes in cells expressing *hBACCS2-IRES-mCherry* or *dmBACCS2-IRES-dOrai-IRES-mCherry* are shown. The cells were light-stimulated once (488-nm laser; illumination indicated by the blue vertical line) at time 0 and the fluorescence changes were measured every 10 s. (**f**) Repeated photoactivation of hBACCS2 and dmBACCS2. hBACCS2 or dmBACCS2 was activated by a short pulse of 488-nm laser light three times (blue vertical lines) at 500-s intervals and the time course of the intracellular Ca^{2+} concentration changes was analysed with Rhod-3/AM. It should be noted that the apparent elevated intracellular Ca^{2+} concentration for *dmBACCS2-IRES-dOrai-IRES-mCherry* during the extended periods in (e) or before the second and third stimuli in (f) is caused by the appearance of bright particles after the induction of high Ca^{2+} signals, which are likely to be artificial signals induced by Rhod-3/AM. Data in (b), (c), (d), (e), and (f) represent means±s.e.m. The cell numbers analysed are indicated in parentheses in (b), (c), (e), and (f). All data were obtained from more than three independent experiments.

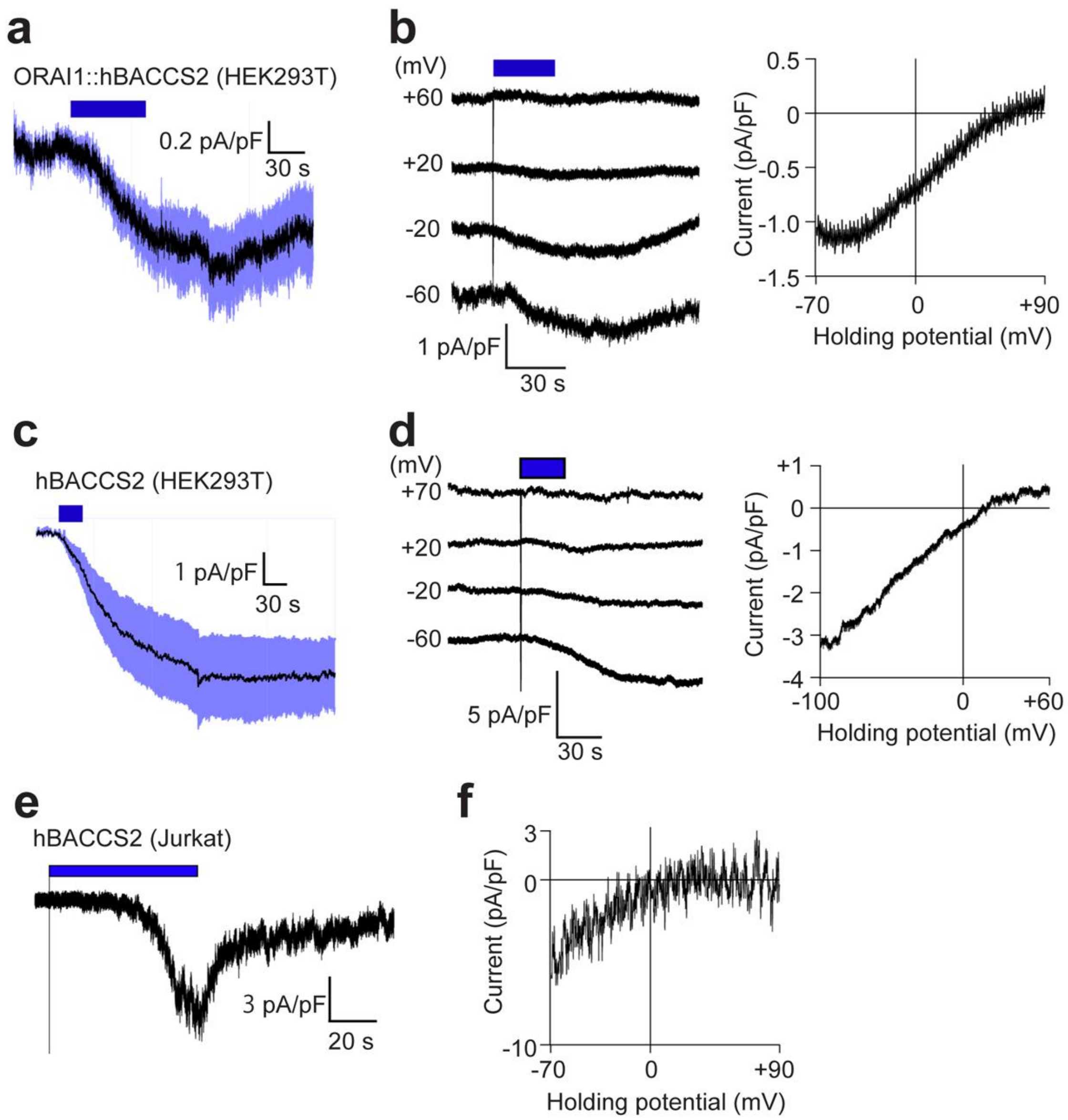

Supplementary Figure 3 | Light stimulation activates calcium conductance in HEK293T cells or Jurkat T cells expressing BACCSs. (**a, b**) Whole-cell inward current responses in HEK293T cells expressing *ORAI1::hBACCS2*. (**a**) Average whole-cell inward current responses with 1 min of blue light stimulation at a holding potential of −60 mV (*n*=6). (**b**) Current responses of a HEK293T cell at various holding potentials (left) and current-voltage (I-V) relationships obtained by a ramp protocol (right). (**c, d**) Whole-cell inward current responses in HEK293T cells expressing *hBACCS2*. (**c**) Average whole-cell inward current responses with 30 s of blue light stimulation at a holding potential of −60 mV (*n*=7). (**d**) Current responses of a HEK293T cell at various holding potentials (left) and I-V relationships obtained by a ramp protocol (right). (**e, f**) Whole-cell inward current responses in Jurkat T

cells expressing *hBACCS2*. (**e**) A current response with 1 min of blue light stimulation at a holding potential of −60 mV. (**f**) I-V relationships obtained by a ramp protocol. The blue bars on the traces in (a), (b), (c), and (e) indicate the timing of the blue light (470 nm) stimulation.

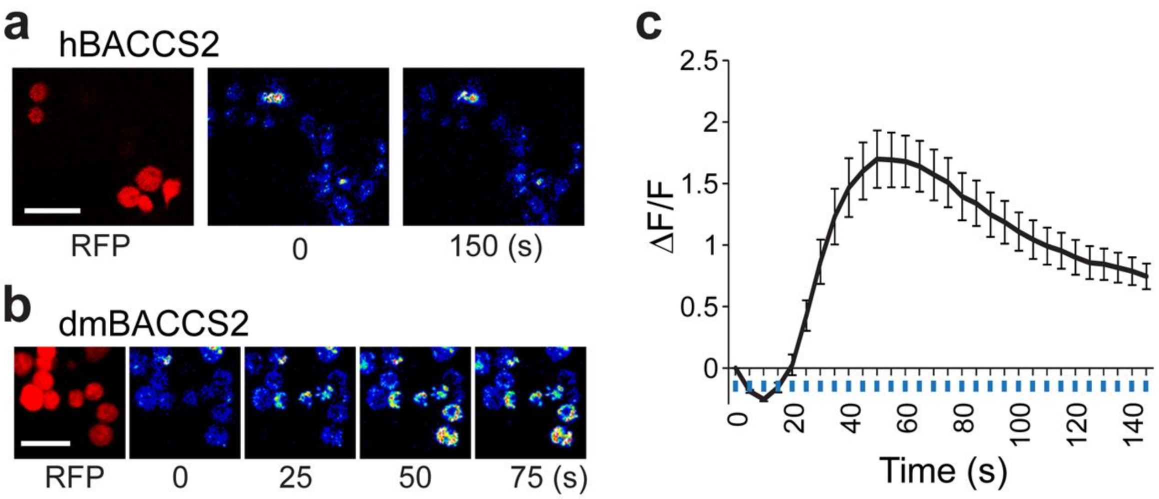

Supplementary Figure 4 | BACCS activation in S2 cells. (**a, b**) Fluo-4/AM fluorescence images of *Drosophila* S2 cells transiently expressing (a) *mRFP::hBACCS2* or (b) *mRFP::dmBACCS2*. Cells were stimulated with a 488-nm laser (0.2 Hz). Scale bars, 20 µm. No Ca^{2+} responses were observed for *mRFP::hBACCS2*-expressing cells (*n*=6 independent experiments), while 27% of *dmBACCS2*-expressing S2 cells showed a Ca^{2+} response. (**c**) Time-course of Fluo-4/AM fluorescence changes in S2 cells transiently expressing *mRFP::dmBACCS2* during the course of photoactivation (488-nm laser; 0.2 Hz; illumination indicated by blue vertical lines). Data represent means±s.e.m (*n*=93 cells in three independent experiments). Pseudocoloured images are shown in (a) and (b) as in Fig. 2a.

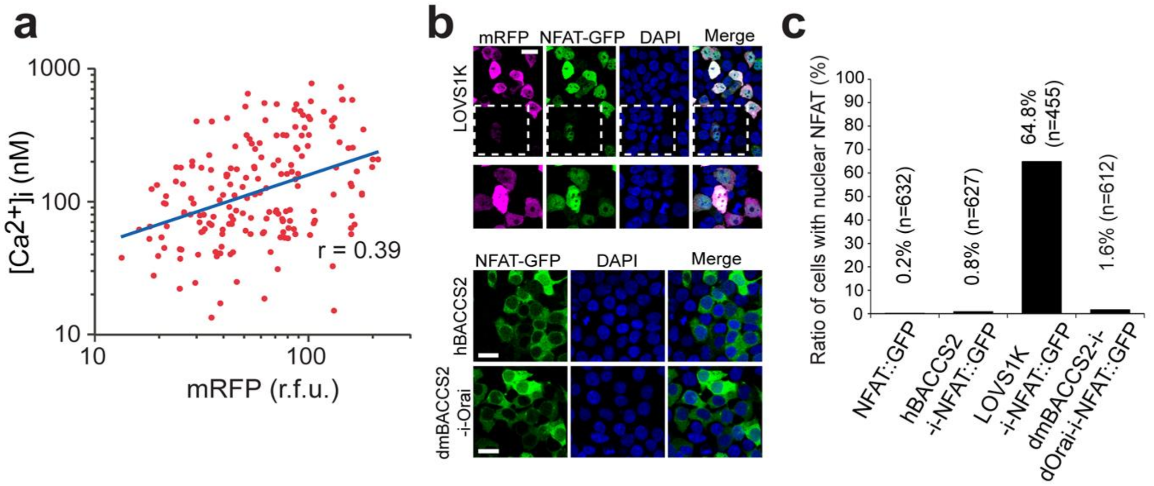

Supplementary Figure 5 | Basal Ca^{2+} levels in HEK293T cells expressing Ca^{2+} photoswitches. (**a**) The expression levels of tdTomato (relative fluorescence units; r.f.u) and basal Ca^{2+} levels in cells expressing *dmBACCS2-IRES-dOrai-IRES-tdTomato* were plotted. A regression line is represented by the blue line. r, Pearson correlation coefficient. Cells expressing a higher level of *dmBACCS2-IRES-dOrai-IRES-tdTomato* tend to show a higher basal Ca^{2+} level. (**b**) Representative images of cells transiently expressing *LOVS1K-IRES-NFAT::GFP*, *hBACCS2-IRES-NFAT::GFP*, or *dmBACCS2-IRES-dOrai-IRES-NFAT::GFP* in the dark. LOVS1K is an mRFP fusion protein. Higher-magnification and brighter views of the boxed areas are shown below for LOVS1K. Cells expressing both high and low levels of LOVS1K show nuclear localization of NFAT in the dark. Scale bars, 20 μm. (**c**) Ratios of cells with NFAT nuclear localization in cells transiently expressing *NFAT::GFP*, *hBACCS2-IRES-NFAT::GFP*, *LOVS1K-IRES-NFAT::GFP*, or *dmBACCS2-IRES-NFAT::GFP*. The ratios of cells with nuclear NFAT among transfected cells were calculated as percentages. Data were obtained from three independent experiments. The exact values and sample sizes (*n*) are shown above the bar graph.

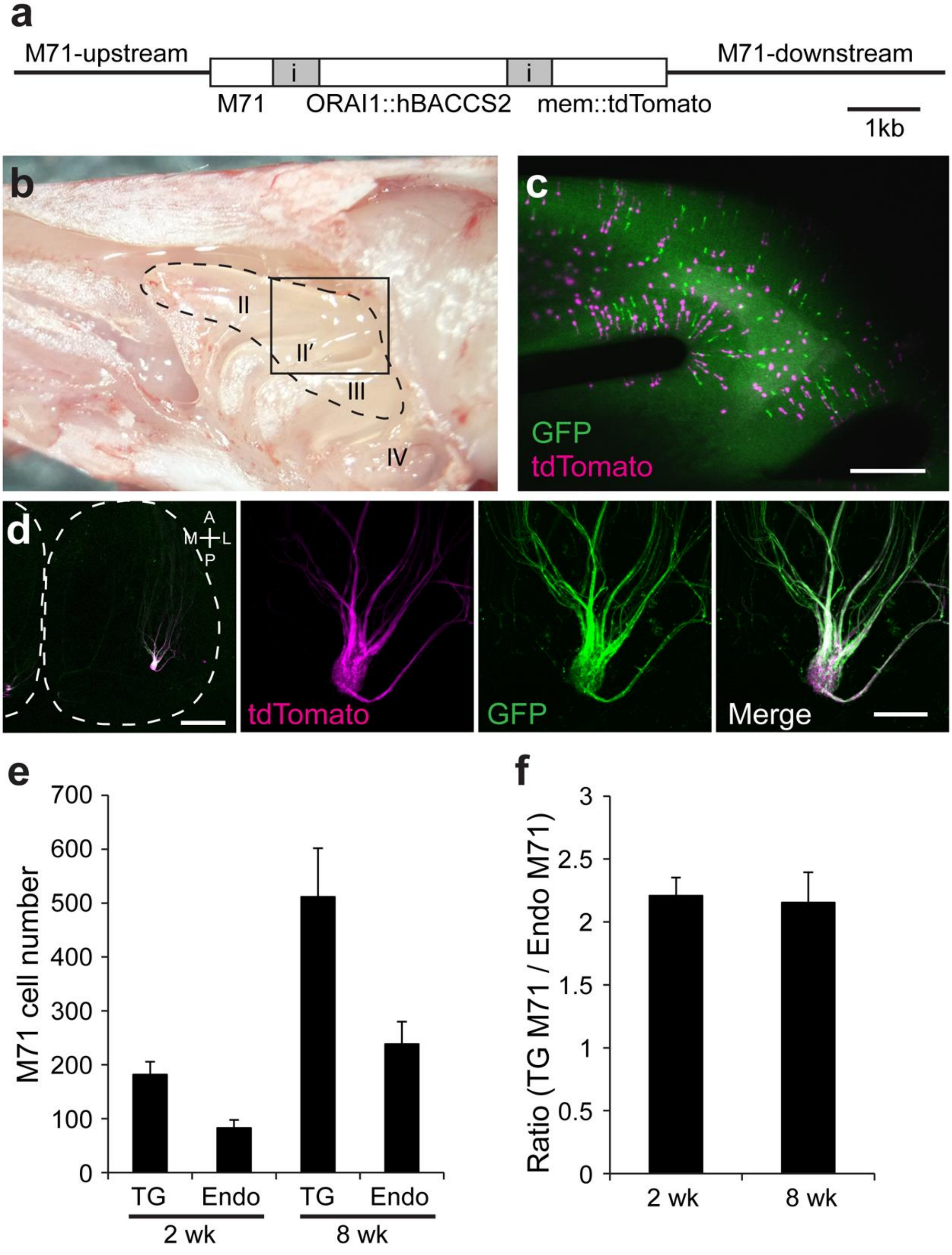

Supplementary Figure 6 | Characterization of BACCS transgenic mice. (**a**) Transgenic construct for expression of ORAI1::hBACCS2 in olfactory sensory neurons (OSNs) expressing the odorant receptor M71. The DNA fragment, *IRES-ORAI1::hBACCS2-IRES-mem::tdTomato*, was inserted into the 3′-UTR of the *M71* coding sequence in a 6.5-kb *M71*

transgenic construct. i, *IRES*. (**b**) Whole-mount lateral view of a mouse olfactory epithelium as a reference. (**c**) Representative fluorescence image of an olfactory epithelium from an 8-week-old *M71-IRES-ORAI1::hBACCS2-IRES-mem::tdTomato* transgenic homozygous *M71-IRES-tauGFP* knock-in mouse[3]. The image corresponds to the boxed area in (b). tdTomato- and GFP-expressing neurons are shown in magenta and green, respectively. Note that the odorant receptor transgene and endogenous gene are known to be expressed in distinct populations of olfactory sensory neurons[4,5]. Scale bar, 200 μm. (**d**) Whole-mount dorsal view of the right olfactory bulb from a 3-week-old *M71-ORAI1::hBACCS2-mem::tdTomato* transgenic homozygous *M71-GFP* knock-in mouse (left panel). The areas of the olfactory bulbs are outlined with dashed lines. Higher-magnification views of the projection site are also shown (right panels). Scale bars, 500 μm in the left panel and 100 μm in the right panel. OSN axons expressing the same type of odorant receptor converge on a specific target site in the olfactory bulb, forming a glomerular structure[6]. It has been reported that neural activity mediated by Ca^{2+}-permeable cyclic nucleotide-gated (CNG) cation channels regulates the glomerular segregation[7]. In the *M71* transgenic mouse, the axons of OSNs expressing the transgene *M71* and endogenous *M71* coalesce into the same glomeruli in the olfactory bulb and the axon terminals are intermingled, suggesting that the basal activity of ORAI1::hBACCS2, if any, is low enough to prevent the glomerular segregation. (**e**) Numbers of cells expressing transgene *M71* and endogenous *M71* in the area defined by the dashed line in (b) at 2- and 8- weeks-old. The data shown are for transgene M71-expressing neurons (TG) in mice at 2 weeks of age (n=8), endogenous M71-expressing neurons (Endo) in mice at 2 weeks of age (n=6), TG in mice at 8 weeks of age (n=6), and Endo in mice at 8 weeks of age (n=6). Data represent means±s.d. (**f**) Ratios of cell numbers for TG to Endo based on the data in (e). The data shown are for mice at 2-weeks-old (mean±s.d., 2.21±0.14; n=8) and 8-weeks-old (2.15±0.24; n=6). There is no significant difference between the ratios of the mice at 2 and 8 weeks of age (P=0.607, t-test). The numbers of OSNs expressing the transgene *M71* and endogenous *M71* increase at the same rate when the mice at 2 and 8 weeks of age are compared, suggesting that additional expression of ORAI1::hBACCS2 in M71 OSNs has little effect on neural survival.

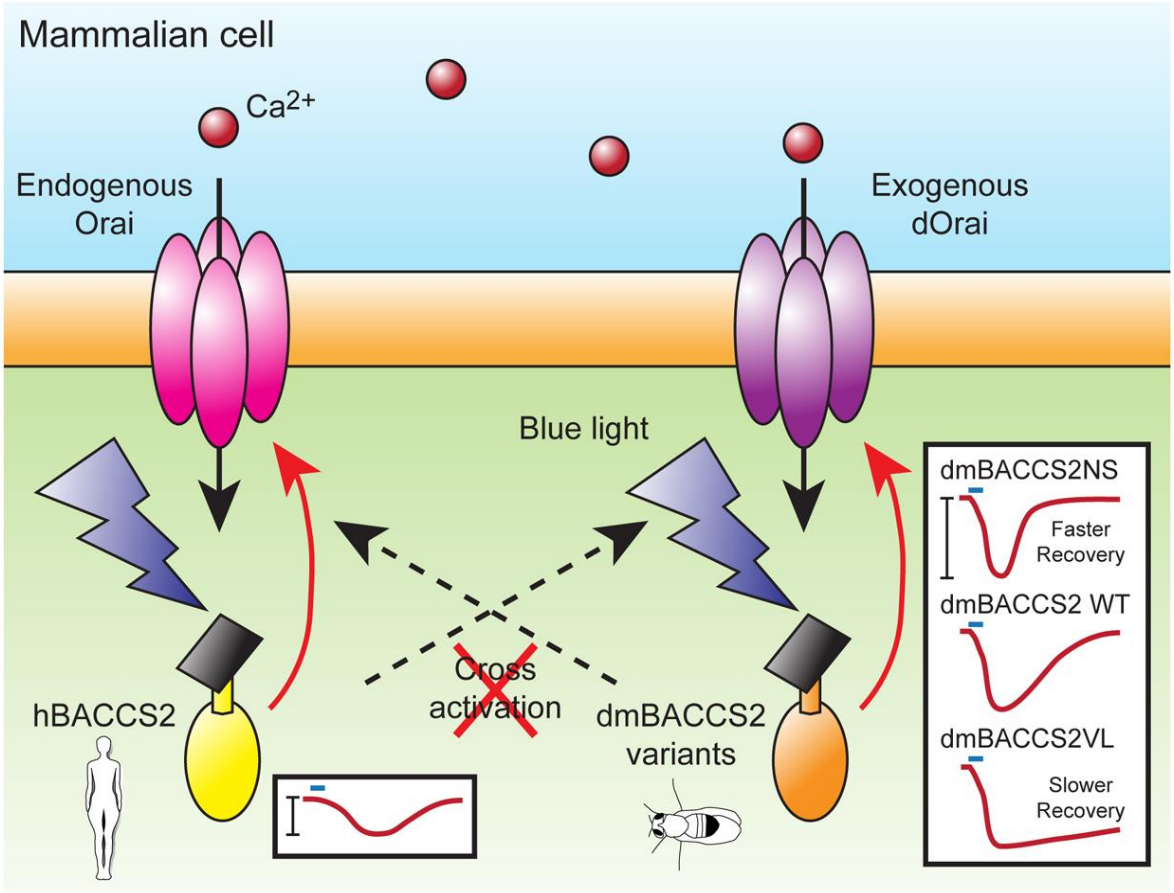

Supplementary Figure 7 | Schematic illustration of the actions of hBACCS2 and dmBACCS2 variants in mammalian cells. In response to blue light, hBACCS2 activates endogenous Orai, while dmBACCS2 variants (dmBACCS2, dmBACCS2NS, dmBACCS2VL) activate exogenous dOrai, resulting in various Ca^{2+} responses.

Construct	A	B
hBACCS1	1.2%	46.2%
hBACCS2	6.9%	98.7%
hBACCS2-i-ORAI1	24.4%	96.6%
ORAI1::hBACCS2	5.8%	98.4%
ORAI1::hBACCS2 (short linker)	3.8%	100%
ORAI1::hBACCS2 (long linker)	15.0%	100%
dmBACCS2-i-dOrai	8.8%	98.0%
LOVS1K	13.8%	95.5%
dmBACCS2NS-i-dOrai	8.2%	73.4%
dmBACCS2VL-i-dOrai	11.7%	90.6%

Supplementary Table 1.

Supplementary data for analysis of Ca^{2+} imaging in HEK293T cells.

A: Ratios of cells excluded from the analysis because of high basal Ca^{2+} levels in the dark. These cells were defined as those with >2× the Fluo-8/AM fluorescence intensity in non-transfected cells.

B: Ratios of cells that responded to blue light. Cells with high basal Ca^{2+} levels were excluded from the analysis.

Supplementary Methods

DNA constructs

All BACCS expression vectors described below were designed to contain the woodchuck hepatitis virus posttranscriptional regulatory element (WPRE) in the 3′ untranslated region (UTR) of the coding sequences to enhance protein expression[8].

To create *NFAT::CFP* and *NFAT::mCherry*, *GFP* was removed from *HA-NFAT (1-460)-GFP* and replaced with *CFP* and *mCherry*, respectively. hBACCS1 was generated by fusing *LOV2-J*α (amino acids 404–538) and *STIM1* (amino acids 347–448) from *PA-Rac1* and human *STIM1* via recombinant PCR. The fusion gene was subcloned into an expression vector derived from pEGFP-N1 to generate phBACCS1, in which the peptide sequences MGPVGGSGGS and GGSGGSGLV were added to the N-terminal and C-terminal ends of the translated protein, respectively. The coding sequence was flanked by an *Xho*I-Kozak sequence and a *Sal*I site. Series of *tdTomato::LOVlit::STIM1* fusion genes and *tdTomato::LOVdark::STIM1* fusion genes were generated by recombinant PCR in a similar manner, except that PA-Rac1-I539E and PA-Rac1-C450A were used as the templates for PCR amplification, respectively, and the amplified fragments were subcloned into the *Bgl*II and *Sal*I sites of ptdTomato, a vector derived from pEGFP-C1 with replacement of *EGFP* by *tdTomato* and addition of WPRE at the 3′-UTR. *hBACCS2* was generated by fusing two *hBACCS1* sequences via recombinant PCR, resulting in the addition of the following peptide sequences: MGPVGGSGGS at the N-terminus; GGSGGSGGGILQSRGGSGGSGGSGS[9] between the two *hBACCS1* sequences; and GGSGGSGLV at the C-terminus. phBACCS2 was generated by replacing *hBACCS1* with *hBACCS2* in phBACCS1. *ORAI1::hBACCS2* was generated by recombinant PCR with insertion of the following linker peptide containing an HA tag between ORAI1 and hBACCS2: RILQSTVPRARDPPVGGYPYDVPDYAGSYPYDVPDYAGSYPYDVPDYAPPVGGSGGS (the HA tag sequence is underlined). The short linker construct of *ORAI1::hBACCS2* was generated by removing the *HA* tag sequence from *ORAI1::hBACCS2*, resulting in a 36-amino-acid shorter version of ORAI1::hBACCS2 (the remaining linker sequence, RILQSTVPRARDPPVGGSGGS). The long linker construct of *ORAI1::hBACCS2* was generated by replacing the *HA* tag sequence with *mCherry* from *ORAI1::hBACCS2*, resulting in a 201-amino-acid longer version of ORAI1::hBACCS2 (the linker sequence, RILQSTVPRARDPPVAT-mCherry-SGLRS). *dmBACCS2* was generated in a similar manner to the generation of *hBACCS2*, except that the sequence derived from human *STIM1* was replaced with the corresponding *Drosophila melanogaster Stim* sequence obtained by PCR

using genomic DNA from *Drosophila* S2 cells and the primers 5′-CTGCAGAAAATCTGCAATCATGGCTTCAATAC-3′ and 5′-GTTCAAGGATCCTCCGGGCAGACCATTGTTGTTCAC-3′. *mRFP::dmBACCS2* was generated by inserting *mRFP* into the *Xho*I and *Bgl*II sites. *dOrai* was amplified by PCR using cDNA from the late third instar of a *D. melanogaster* mutant strain (*yellow*, *white*) and the primers 5′-GGTGACTCGAGCCACCATGTCTGTGTGGACCACGGC-3′, and 5′-TCACCGTCGACCTAGACAATGTCCCCGGATG-3′. dmBACCS2NS and dmBACCS2VL were generated by recombinant PCR starting with dmBACCS2 to introduce the mutations N425S and V416L into two LOV2 domains of each construct, respectively. A *LOVS1K* coding sequence flanked by an *Xho*I-Kozak sequence and a *Sal*I site was generated by PCR from *LOVS1K* and subcloned into the *Xho*I and *Sal*I sites of phBACCS1, replacing the *hBACCS1* coding sequence. All internal ribosomal entry site (*IRES*)-containing cassettes for *tdTomato*, *mem::tdTomato*, *GFP*, *mCherry*, *iRFP*, *mem::iRFP*, *NFAT::mCherry*, *NFAT::GFP*, *ORAI1*, *dOrai*, and *ORAI1::hBACCS2* were generated by recombinant PCR to place each gene exactly at the translation start site, and subcloned into the *Xho*I and *Sal*I sites of pBluescript SK(+). For all expression vectors containing *IRES*-cassette(s), the *Xho*I–*Sal*I fragment of the *IRES*-cassette was inserted into the *Sal*I site at the 3′-UTR of each gene. For expression vectors in S2 cells, genes were inserted into the *Not*I and *Xba*I sites of pAc5.1/V5-HisA (Invitrogen).

For the transgenic mouse construct, the *Not*I–*Nhe*I fragment was removed from M71-TV to shorten the construct. The *IRES-ORAI1::hBACCS2-IRES-mem::tdTomato* cassette was inserted into the *Pac*I site located at the 3′-UTR of the odorant receptor gene *M71* in the shortened M71-TV, resulting in *M71-IRES-ORAI1::hBACCS2-IRES-mem::tdTomato*.

For the adenovirus vector, *hBACCS2-IRES-mem::tdTomato* was inserted into the *Swa*I site of the cosmid vector pAxCAwtit (TaKaRa).

All coding regions were verified by sequencing.

Supplementary References

1. Harper, S. M., Christie, J. M. & Gardner, K. H. Disruption of the LOV-Jalpha helix interaction activates phototropin kinase activity. *Biochemistry* **43,** 16184–92 (2004).

2. Salomon, M., Christie, J. M., Knieb, E., Lempert, U. & Briggs, W. R. Photochemical and mutational analysis of the FMN-binding domains of the plant blue light receptor, phototropin. *Biochemistry* **39,** 9401–10 (2000).

3. Bozza, T., Feinstein, P., Zheng, C. & Mombaerts, P. Odorant receptor expression defines functional units in the mouse olfactory system. *J. Neurosci.* **22,** 3033–43 (2002).

4. Serizawa, S. *et al.* Mutually exclusive expression of odorant receptor transgenes. *Nat. Neurosci.* **3,** 687–93 (2000).

5. Vassalli, A., Rothman, A., Feinstein, P., Zapotocky, M. & Mombaerts, P. Minigenes impart odorant receptor-specific axon guidance in the olfactory bulb. *Neuron* **35,** 681–96 (2002).

6. Sakano, H. Neural map formation in the mouse olfactory system. *Neuron* **67,** 530–42 (2010).

7. Serizawa, S. *et al.* A neuronal identity code for the odorant receptor-specific and activity-dependent axon sorting. *Cell* **127,** 1057–69 (2006).

8. Zufferey, R., Donello, J. E., Trono, D. & Hope, T. J. Woodchuck hepatitis virus posttranscriptional regulatory element enhances expression of transgenes delivered by retroviral vectors. *J. Virol.* **73,** 2886–92 (1999).

9. Li, Z. *et al.* Graded activation of CRAC channel by binding of different numbers of STIM1 to Orai1 subunits. *Cell Res.* **21,** 305–15 (2011).
